# Fluorescently Labelled Polypropylene as a Model Microplastic for Cellular Imaging

**DOI:** 10.1039/d6py00089d

**Published:** 2026

**Authors:** Alexander Evans, Ríona M. Devereux, Zoë R. Turner, Angela J. Russell, Dermot O’Hare

**Affiliations:** Chemistry Research Laboratory, Department of Chemistry, University of Oxford, 12 Mansfield Road, OX1 3TA Oxford, UK

**Keywords:** Microplastics, polymer, fluorescent polypropylene, cells, imaging

## Abstract

Polypropylene (**PP**) is the second most produced plastic globally and is environmentally pervasive in the form of microplastics (MP). The cellular localisation and fate of MPs within biological systems remain poorly understood. We present a method for the covalent functionalisation of PP with the fluorescent label, rhodamine B (**Rb**), and its subsequent MP evaluation in a human embryonic kidney model cell line (HEK293T). **Rb** was successfully coupled into PP (**PP**_**Rb**_) under Steglich esterification conditions. **PP**_**Rb**_ is stabile under physiological pH and so can be utilised *in vitro*, offering a significantly improved MP model to conventional staining methods that can definitively locate MP in human cells. Cellular uptake of **PP**_**Rb**_ was rapid (≤ 1 h), non-toxic up to 70 µM, and was not observed to localise within the nucleus. Increased mean lysosomal fluorescence intensity suggested an endosomal uptake mechanism followed by significant clearance over a 48 h period. This approach provides a robust platform for definitive microplastic tracking, enabling reliable, standardised studies of polymer–cell interactions across biological systems.

## Graphical abstract

**Toc Graphic:**
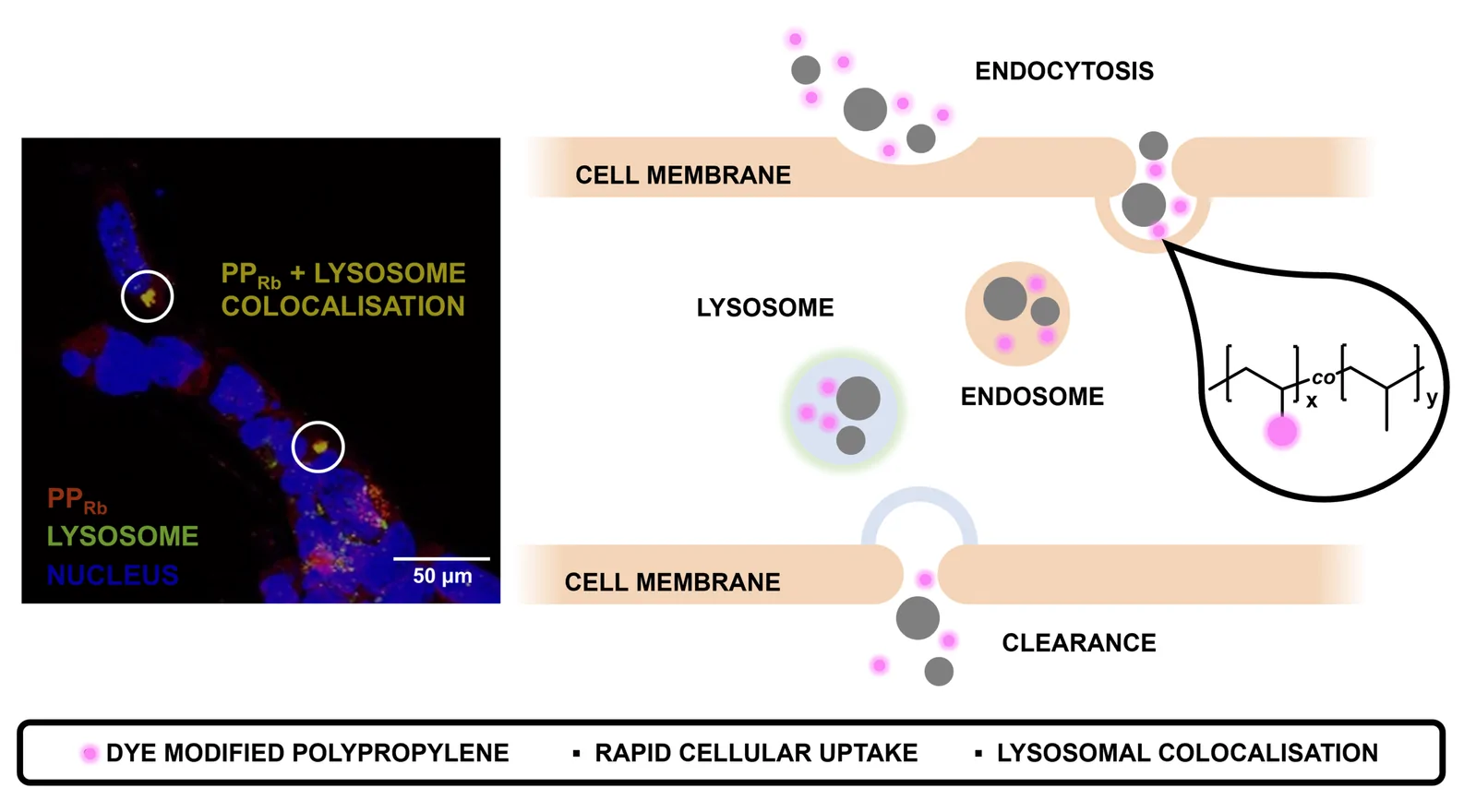

## Introduction

Commercially, polyolefins (PO), namely polypropylene (PP)^1^ and polyethylene (PE),^2^ are synthesised at scale to meet the needs of a broad array of applications; with PP accounting for 17% of global plastic production.^3^ Desirable and tuneable properties, such as mechanical strength or creep resistance, as a result of branching, molecular mass (*M*_n_), and dispersities (*Ð*_M_), coupled with low production costs are amongst the driving forces behind their widespread utility.^4^ Although PP is recyclable, a significant proportion enter landfill or the environment.^5–7^ An abundance of PP waste is converted to microplastics (MP),^8^ or indeed nanoplastics,^9^ *via* slow degradation due to (a) biotic factors.^7^ Detection of PO MP is widely reported, and the MP highly prevalent.^10, 11^ Plastic debris is an environmentally persistent and complex contaminant (e.g. varying in size, morphology, chemical constituency),^12^ whilst MP detection remains challenging.^13, 14^ Consequently, recent legislative efforts have been introduced in Europe to curtail the prevalence of single use plastics given the emergence of MP as a pressing environmental concern due to their ubiquity, potential ecological ramifications, and associated risk.^10, 14–17^

The impact of MP on macroscopic organisms have been extensively studied with recent reports evaluating effects on mice,^18, 19^ fish,^20–22^ and humans.^23^ Reports detailing MP entry into aquatic and terrestrial food chains *via* biomagnification and bioaccumulation,^12, 24–26^ and detection in fish, birds, and mammals feeding on aquatic organisms or living in aquatic environments has been the subject of further investigation.^22, 27^ Perturbation to ecological processes may align with other anthropogenic pollutants despite the lack of lethality; reduced fecundity and growth at the first trophic level may cause persistent reduction of biomass.^28, 29^ Additionally, investigations into which animals can ingest MP and the mechanism by which this occurs has been reported.^30, 31^ However, the marked chemical inertness severely hampers evaluation on living species; the incorporation of a fluorescent probe circumvents MP detection issues in organic tissues.^32–37^

However, the effects on single-celled organisms, which constitute fundamental components of aquatic ecosystems, remain relatively underexplored. These minute organisms, encompassing diverse taxa such as phytoplankton and protozoa, play pivotal roles in nutrient cycling and food web dynamics.^29, 38–40^ The ingestion of microplastics by single-celled organisms can lead to various detrimental outcomes, including altered feeding behaviours, impaired metabolic functions, and compromised reproductive success.^29, 38–40^ Furthermore, the sorption of toxic chemicals onto microplastics introduces an additional layer of risk, as the bioavailability of these pollutants increases upon ingestion by these microorganisms,^13, 16, 28, 33, 41^ although disputed.^10^ Despite the limited research in this area, the potential for cascading effects throughout aquatic ecosystems underscores the urgency to comprehensively elucidate the consequences of microplastic exposure on single-celled organisms. Ascertaining an understanding of the posed risk is crucial for the educated development of policy. Current methods for evaluating the role of MP with single-celled organisms predominantly utilise staining,^42^ due to the relative synthetic simplicity,^43^ although some dyes are covalently bound.^37^ *In vitro* assays on mice^44^ and human^45^ cell lines utilise polystyrene (PS) for this purpose. However, this methodology has drawbacks, primarily leaching of free dye, potentially leading to the presence of artefacts, confounding interpretation of the results.^46–48^ Subsequently, reports with claims of polymer sample purity, or indeed covalent coupling of dye units to the polymer chain, are not supported by adequate rigorous characterisation data leading to the ambiguous interpretation of results.

In the field of mechanical recycling, separation techniques have been extended to include fluorescent labelling, enabling separation by specific polymeric material;^49–51^ recent reports utilise fluorescently tagged PE in this way.^52^ Notably, recycling process typically involve high temperatures and consequently careful selection of incorporated spectroscopic markers is required to avoid degradation.^52^ To the best of our knowledge, recent literature presents a few examples of synthesised *isotactic*-PP copolymers containing fluorescent units, and only work by Pragliola *et al*. has evaluated them as a fluorescent marker of PP thus far.^32, 53^ Fluorescent probe coupled PP may be used to mimic the *in vivo* behaviour of PP-derived MP pollutants on a cellular level.

Several Norwegian institutions and initiatives have pioneered extensive studies in evaluating MP research;^54^ despite a plethora of published articles, it is posited that a lack of standardisation and validated methods, particularly for the smallest plastic particles, hamper conclusive and harmonised research.^55^ Given the interdisciplinary nature of the problem, it is well within the remit of polymer scientists to design, synthesise, and provide specific model polymers for MP analysis in collaboration with relevant academic disciplines. We have recently reported a general two-step methodology to functionalise PP, creating a wide range of covalent linkages, something traditionally difficult to achieve in a controlled manner.^56^ Consequently, we present a method for the covalent fluorescent labelling of rhodamine B (**Rb**) functionalised PP, **PP**_**Rb**_, and subsequent MP evaluation in a human kidney cell line. We believe that this work provides a new methodology for covalently bound fluorescent tags on a PO system which allows confident analysis of any polymer–cell interaction. This has the potential to offer a harmonised approach for the study of related polymeric materials and enable conclusive evaluation of the interaction of such ubiquitous materials within cells and organisms.

## Results and Discussion

### Synthesis and Characterisation of PP_Rb_

**Figure 1:**
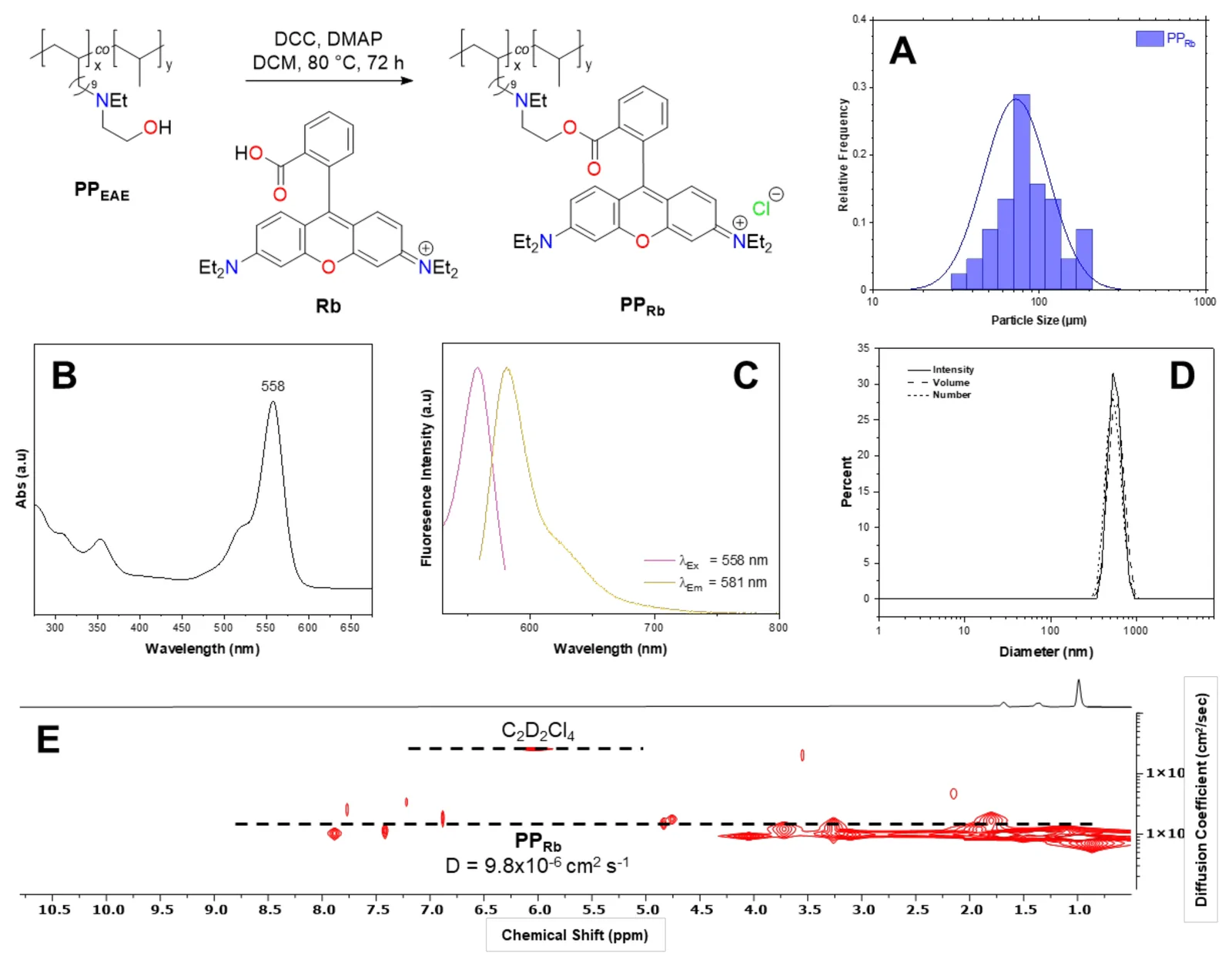
Scheme Synthesis of **PP**_**Rb**_ under Steglich esterification conditions, (**A**) Solid particle size distribution histogram as determined by SEM (Figure S9), (**B**) UV-Vis spectrum of **PP**_**Rb**_ solution (0.5 mg mL^–1^, chloroform), (**C**) Fluorescence spectrum of **PP**_**Rb**_ solution (0.5 mg mL^–1^, chloroform), (**D**) DLS analysis of **PP**_**Rb**_ solution (1 mg mL^–1^, toluene), and (**E**) DOSY ^1^H NMR spectrum (500 MHz, C_2_D_2_Cl_4_, 393 K) of **PP**_**Rb**_.

**PP**_**Rb**_ was synthesised *via* the quantitative transformation of previously reported alcohol-functionalised PP copolymer (**PP**_**EAE**_) under Steglich esterification conditions (DMAP 0.3 eq., DCC 2 eq., DCM, 80 ºC, 72 h) with Rb (2 eq.) (Figure 1).^56, 57^

Following extensive purification by water Soxhlet extraction, **PP**_**Rb**_ with 1.87 mol% covalently-bound dye incorporation was isolated and fully characterised. Key ^1^H NMR spectroscopic handles evidence the presence of dye; six aromatic resonances between 6.37–7.91 ppm integrate appropriately (Figure S1). Convection compensated diffusion experiments support the conclusion that **Rb** is bound to a higher molecular weight species with a diffusion coefficient of 9.8 × 10^–6^ cm^2^ s^–1^ (Figure 1E), which is comparable to the parent copolymer (*ca*. 9.9 × 10^–6^ cm^2^ s^–1^) and considerably different from the free dye (*ca*. 6.2 × 10^–6^ cm^2^ s^–1^) as tracked by aromatic protons and distinct quartet assigned to -NC***H***_2_CH_3_ (3.44 ppm) (Figure S1–2). **PP**_**Rb**_ solution in chloroform (0.5 mg mL^–1^) displays an excitation wavelength (λ_Ex_) at 558 nm by ultraviolet-visible (UV-Vis) spectroscopy (Figure 1B). Significantly, the polymer was also shown to be fluorescent with an emission wavelength (λ_Em_) at 581 nm (Figure 1C). Crucially, λ_Ex_ and λ_Em_ are distinctly altered when compared to derivative **Rb** (λ_Ex_ = 569 nm, λ_Em_ = 592 nm) in comparable molar concentration of fluorophore in chloroform (Figure S8).

The polymer was further characterised by Fourier transform infrared spectroscopy (*ν* = 1736 cm^–1^ (C=O) and 808 cm^–1^ (C=C_*Ar*_)), differential scanning calorimetry (*T*_m_ = 113 °C), and evidenced a single decomposition step (*T*_max_ = 431 °C) by thermogravimetric analysis (Figure S6). Multiple complementary techniques were employed to assess polymer particle size, as variations in measurement conditions can substantially influence the observed dimensions. *M*_n_ analysis by size exclusion chromatography (SEC) was unsuccessful, with **PP**_**Rb**_ not eluting within the calibration window; however, theoretical molar mass (*M*_n, th_) is predicted *ca*. 14 700 g mol^–1^ based on parent copolymer experimental molar mass (*M*_n, SEC_ = 13 800 g mol^–1^) (Figure S6). Since the Mark Houwink correlation is not known for functionalized PP, and the presence of a polar group is likely to affect the radius of gyration in solution, but it is reasonable to assume that the backbone chain length is unchanged during post polymerization modification.^58^ Dynamic light scattering (DLS) suggests that **PP**_**Rb**_ aggregates in the solution state, common for amphiphilic copolymers and potentially induced by the ionic nature of the fluorophore.^59^ **PP**_**Rb**_ in toluene solution (1 mg mL^–1^) displayed good agreement between number, volume, and intensity values for hydrodynamic diameter (*D*_h_), *ca*. 570 nm, and increased from the parent copolymer *ca*. 140 nm, supporting a micellar arrangement in solution with increasing hydrophilic character (Figure 1D, Figure S7, and Table S1). It is worth noting that this may subsequently affect the permeability of **PP**_**Rb**_ towards the cell membrane; however, existing *in vitro* experiments employ charged, colloidally stable PS particles^44^ and we believe our approach offers an alternate with significantly reduced impact (*vide infra*). Additionally, some **PP**_**Rb**_ was cryofractured (^**c**^**PP**_**Rb**_) in an attempt to simulate accelerated weathering (Figure S9 and S10); such that the irregularities in size and shape of MP found within the environment could be rudimentarily assessed.^32^

### Cellular Studies

Incorporating fluorophores directly into MP models marks a critical advancement in visualising and understanding MP interactions within biological systems. Fluorophore incorporation on PP enabled an *in vitro* investigation in a human embryonic kidney (HEK293T) model cell line where **PP**_**Rb**_ is utilised as a model for MP, overcoming imaging issues suffered by traditional polyolefins. Through the covalent linkage of the dye moiety, it can be concluded that any observations arise due to the presence of dye-bound polymer and not through leaching; other methods previously reported suffer from this drawback.^42, 48, 60^ A control experiment was performed to measure polymer-dye stability using a citrate-phosphate buffer solution, mimicking the acidic lysosomal compartments (pH 4, 38 °C), and detailed in the Supporting Information.^61^ Detected fluorescence correlated to a maximum hydrolysis of 1.6% over 3 days, and deemed to not be statistically significant, suggesting minimal degradation in a physiologically relevant pH range (Figure S17).

HEK293T cells were chosen as the model cell line to investigate uptake and localisation *via* confocal microscopy. The primary entry points for MPs into the human body are ingestion, inhalation, and dermal contact.^62, 63^ Once internalised, MPs can enter the circulatory system, potentially accumulating in organs such as the gut, liver, and kidneys.^64^ Given the kidney’s role in filtering and detoxifying various substances, including environmental contaminants like MPs, the HEK293T cell line serves as a relevant model for examining cellular defence mechanisms in response to MPs.^65^

**Figure 2:**
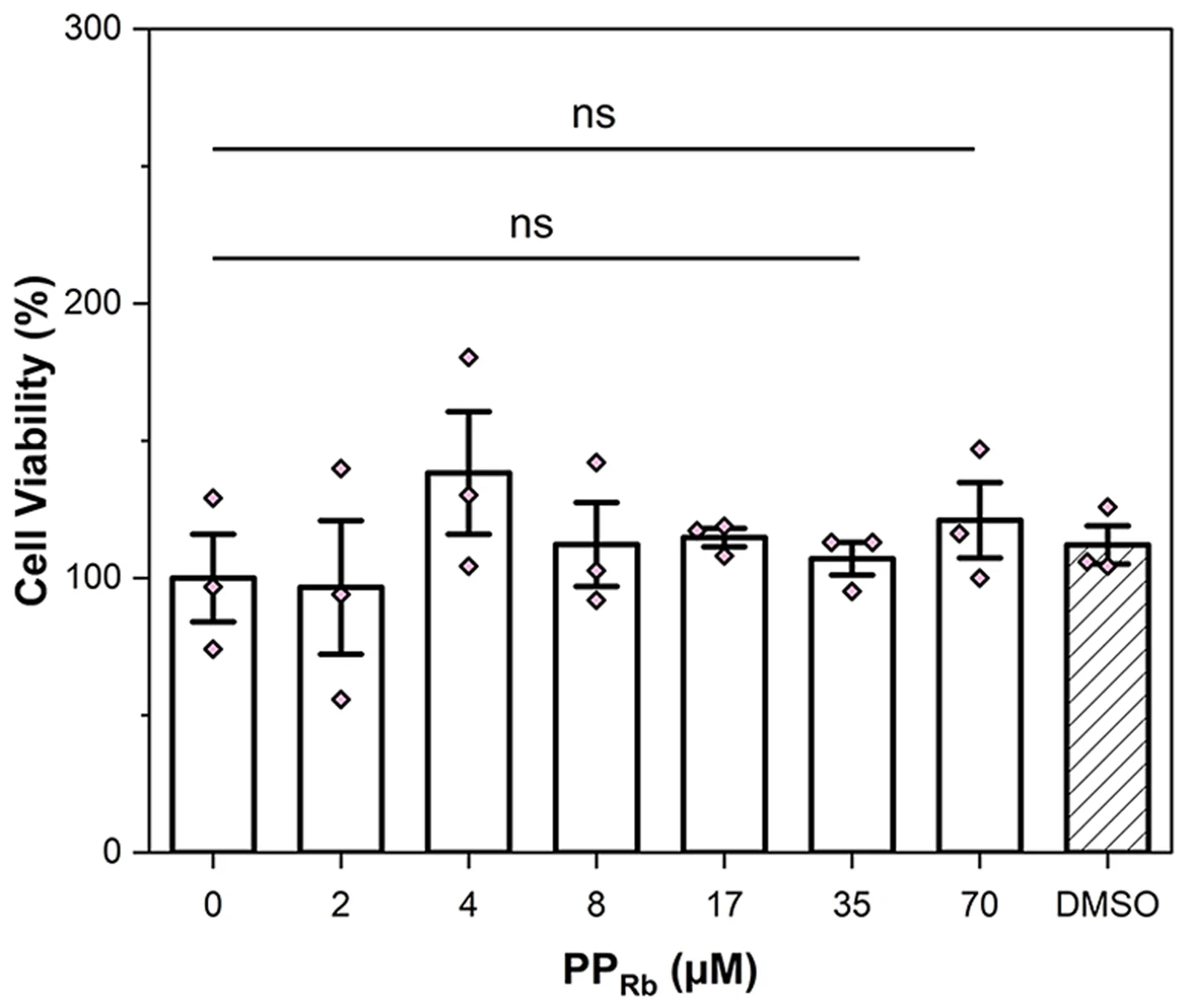
*In vitro* cytotoxicity assessment of **PP**_**Rb**_ in HEK293 cells using the AlamarBlue™ Cell Viability Assay. Cell viability (%) in response to various concentrations of **PP**_**Rb**_ (0–70 µM) after 24 h of treatment, as compared to the DMSO control group. Data are represented as mean ± standard error of the mean (*n* = 3). Brown-Forsythe and Welch one-way ANOVA, non-parametric (*ns* = *p* > 0.05).

The acute cytotoxicity of **PP**_**Rb**_ was assessed *in vitro* using the AlamarBlue™ Cell Viability Assay in HEK293T cells. Cells were exposed to a range of [**PP**_**Rb**_] for 24 h, with cell viability measured across the concentration gradient. No significant cytotoxic effects were observed up to the highest concentration tested (**PP**_**Rb**_ = 70 µM), as compared to the control group (**PP**_**Rb**_ = 0 µM) (Figure 2). Additionally, bright-field microscopy of cells treated with 35 µM **PP**_**Rb**_ for 24 hours revealed no signs of cell death or structural abnormalities relative to untreated control cells (Figure 2 and S13). It is worth noting that **PP**_**Rb**_ was rigorously purified prior to cell treatment. In environmental contexts, however, MPs often act as sorbents, potentially concentrating harmful and toxic materials on their surfaces, which may influence their biological effects.^33, 66–68^

**Figure 3:**
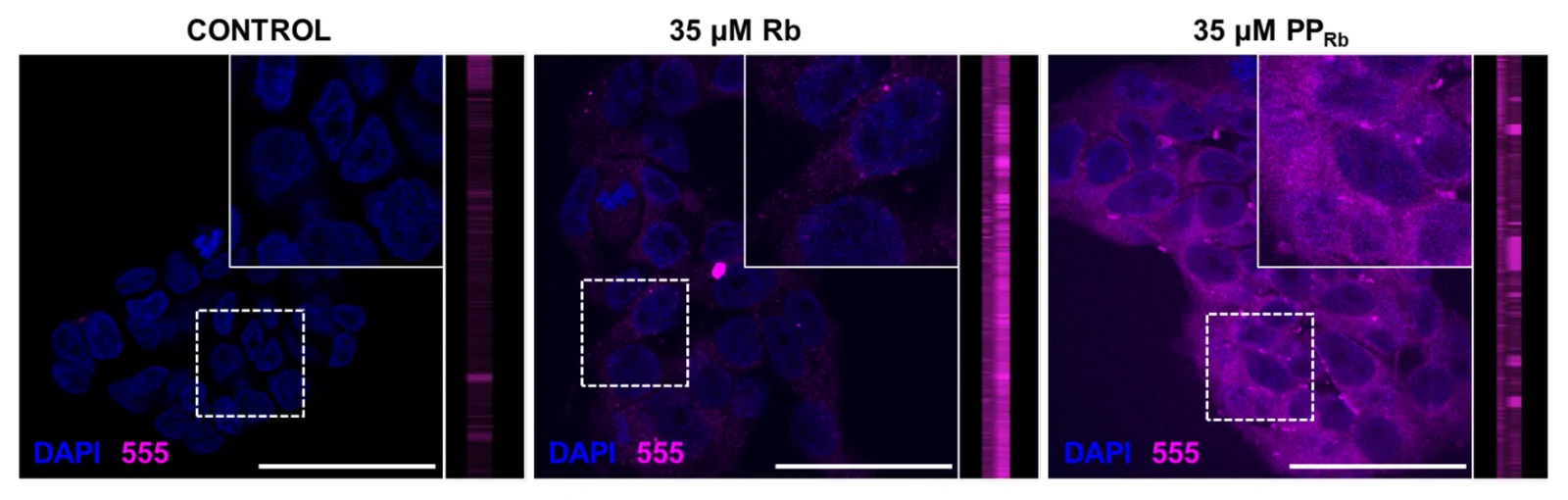
Confocal microscopy images showing the cellular localisation of dye-bound polymer **PP**_**Rb**_ and free **Rb** in HEK293T cells. Cells were incubated with either 35 µM of free **Rb** or **PP**_**Rb**_ for 1 h and compared with untreated control cells. The images display fluorescence representing the presence of **Rb** or **PP**_**Rb**_ (magenta) and DAPI nuclear staining (blue). The inset (top right) presents a (2×) zoomed-in view highlighting the compartmentalisation of **Rb** or **PP**_**Rb**_. Z-stack panels (side view) illustrate the spatial distribution of fluorescence within the cells. Scale bars = 50 µm.

To assess the uptake and localisation of **PP**_**Rb**_ *in vitro*, HEK293T cells were incubated with either 35 µM of unbound fluorophore (**Rb**) or fluorophore-bound **PP**_**Rb**_ for 1 h and compared with untreated control cells (Figure 3). A detectable signal was observed in the 555 nm channel for both **Rb** and **PP**_**Rb**_, with minimal background fluorescence in the negative control group. Notably, cells treated with **PP**_**Rb**_ exhibited a higher signal intensity compared to those treated with free **Rb**, indicating a consistent cellular uptake of **PP**_**Rb**_ without removal upon washing. The 1 h treatment period demonstrates rapid cellular uptake and accumulation of **PP**_**Rb**_ within HEK293T cells. The dye-bound polymer, **PP**_**Rb**_, exhibits localisation within cells due to its polymeric structure, in contrast to the free **Rb** dye, which results in a more diffuse cellular distribution. Accumulation of **PP**_**Rb**_ within cells was evident, as confirmed by z-stack imaging (Figure 3), highlighting the polymer’s capacity for localised uptake and retention compared with the less structured, widespread localisation pattern observed with free **Rb**. The different observed cellular behaviours of both **Rb** and **PP**_**Rb**_ are consistent with the absence of ester hydrolysis and dye liberation from **PP**_**Rb**_ (Figure S17).^69^

**Figure 4:**
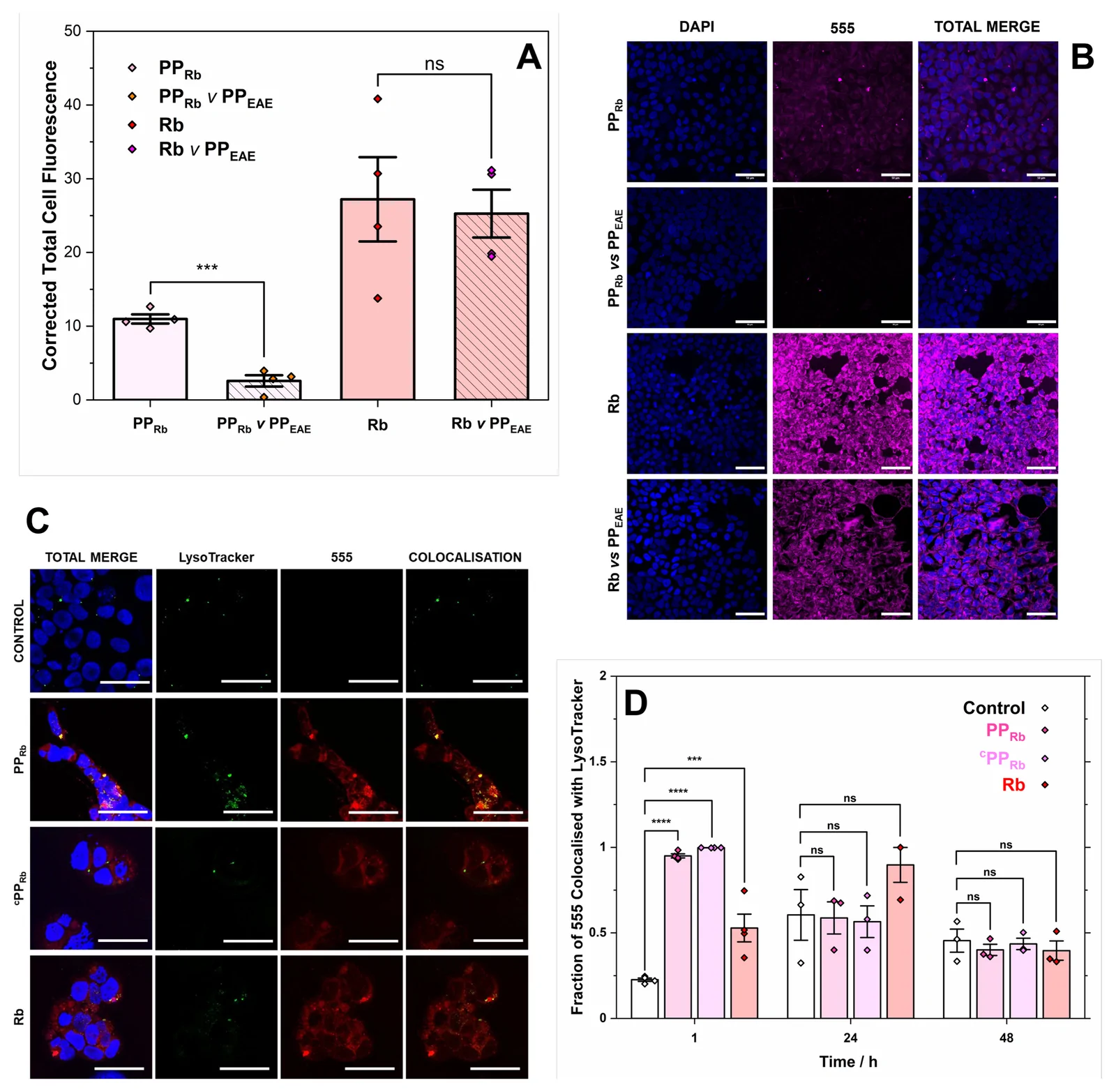
(**A**/**B**) Comparison of Corrected Total Cell Fluorescence at 555 nm for **PP**_**Rb**_, the competition condition (**PP**_**Rb**_ *vs* **PP**_**EAE**_), free **Rb**, and **Rb** co-incubated with **PP**_**EAE**_ (**Rb** *vs* **PP**_**EAE**_) in a fluorescence competition experiment at 24 h timepoint (*n* = 3). (**A**) Quantification showed a significant reduction in fluorescence for the competition condition **PP**_**Rb**_ *vs* **PP**_**EAE**_ compared to **PP**_**Rb**_, indicating that fluorescent localisation is outcompeted by the parent compound **PP**_**EAE**_ at 24 h. The fluorescence of free **Rb** remains unchanged with cotreatment with **PP**_**EAE**_ (**Rb** *vs* **PP**_**EAE**_). (**B**) Confocal microscopy images showing nuclear staining with DAPI (blue), **Rb** fluorescence (555 nm), and merged channels for **PP**_**Rb**_, **PP**_**Rb**_ vs **PP**_**EAE**_, **Rb**, and **Rb** vs **PP**_**EAE**_ conditions at 24 h timepoint. Scale bars 50 µm. (**C**/**D**) Quantification of colocalization of **PP**_**Rb**_, ^**c**^**PP**_**Rb**_, and free **Rb** with lysosomes in HEK293T cells at various time points. (**C**) Confocal microscopy images showing lysosomal staining with LysoTracker (green), nuclear staining with DAPI (blue) and 555 channel (red) in control cells and cells treated with 35 µM of **PP**_**Rb**_, ^**c**^**PP**_**Rb**_, or free **Rb** for 1 h. Scale bars = 50 µm. (**D**) The fraction of 555 colocalization with LysoTracker was quantified for each treatment group (See also Figures S14-15). (**A**/**D**) Data are represented as mean ± standard error of the mean (n = 3). Brown-Forsythe and Welch one-way ANOVA, non-parametric (*** *p* < 0.001, ****p < 0.0001, and *ns* = *p* > 0.05 compared to control).

After 24 h, cellular localisation of **PP**_**Rb**_ is reduced compared to the 1 h time point, suggesting a potential decrease in intracellular retention (Figure 4B). This reduction in cellular localisation may indicate that **PP**_**Rb**_ is being actively removed from the cell through a cellular clearance pathway, possibly *via* an endosomal recycling pathway, although a metabolic degradation pathway cannot be ruled out.^70^

A competition assay was conducted to assess the influence of the parent alcohol-functionalised copolymer, **PP**_**EAE**_, on the cellular localisation of **PP**_**Rb**_ after 24 h (Figure 4A-B). The results indicated that the parent polymer directs cellular localisation of **PP**_**Rb**_, as fluorescence was significantly decreased upon co-incubation with 35 µM **PP**_**EAE**_. In contrast, the localisation of **Rb** remained unaffected by the presence of **PP**_**EAE**_, supporting the conclusion that **PP**_**EAE**_ and **PP**_**Rb**_ localise to the same cellular compartments, while **Rb** does not. This further supports the limited impact of the charged fluorophore driving cellular uptake, trafficking and compartmentalisation, strengthening the role of **PP**_**Rb**_ as a model MP. Notably, this competitive reduction in fluorescence was not observed with the **Rb** fluorophore alone, underscoring that **PP**_**EAE**_, and not **Rb**, modulates the intracellular dynamics of **PP**_**Rb**_ localisation.

Furthermore, a sample of **PP**_**Rb**_ was subjected to cryogenic fracturing to produce ^**c**^**PP**_**Rb**_, a model intended to simulate accelerated environmental degradation.^32^ However, data showed no significant difference in cellular localisation between ^**c**^**PP**_**Rb**_ and the original **PP**_**Rb**_ sample (Figure S11). Importantly, across all timepoints, there was no detectable co-localisation of **PP**_**Rb**_ and nuclear stain, DAPI (Figure S11). Excluding certain molecules or structures from the nucleus can prevent potential disruptions to nuclear architecture or DNA stability, safeguarding essential cellular processes.^71^

It was hypothesised that, due to the polymer size and relatively low *M*_n_, the polymer, or any nanoparticles formed through self-assembly, would enter the cells through an energy-dependent trafficking mechanism *via* an endosomal pathway.^72^ It is, therefore, reasonable to infer possible trafficking of **PP**_**Rb**_ to the lysosomes upon cellular entry. To investigate this, HEK293T cells were stained with LysoTracker, an acidic vesicle-labelling dye, and incubated with **PP**_**Rb**_ for 1, 24, and 48 h (Figure 4C, S14-16). The mean LysoTracker fluorescence intensity for **PP**_**Rb**_, ^**c**^**PP**_**Rb**_ and free **Rb** increased relative to the control at 1 and 24 h, suggesting a potential endosomal uptake mechanism. By 48 h, however, the mean fluorescence intensity of LysoTracker in the **PP**_**Rb**_ and ^**c**^**PP**_**Rb**_ treated cells returned to control levels, while fluorescence for free **Rb** remained elevated. Following this, the colocalization of the 555 channels with LysoTracker was investigated for **PP**_**Rb**_, ^**c**^**PP**_**Rb**,_ and **Rb** (Figures 4C-D and S14-15). Incubation of **PP**_**Rb**_ and ^**c**^**PP**_**Rb**_ showed a significant fraction of channel overlap at 1 h, confirming colocalization of the polymer with lysosomes *in vitro* at this early timepoint. However, this overlap was insignificant at the 24 and 48 h timepoints. These colocalization results further support a lysosomal uptake mechanism. Free **Rb** showed no significant overlap at all tested timepoints, suggesting that the increase in lysosomes may not be directly associated with free **Rb** and could be a secondary effect of its incubation. These observations and earlier dosing data showing reduced **PP**_**Rb**_ uptake by 24 h (Figure 4B) suggest that cells may actively work to clear the polymer over time.

### Conclusionary Remarks

Polypropylene with covalently-bound fluorescent rhodamine B tag, **PP**_**Rb**_, was synthesised and characterised for *in vitro* studies against a HEK293T cell line and evaluated by confocal microscopy. The two-step copolymerization – post-modification strategy employed is facile and, importantly, allows preparation of rigorously purified model polymer samples that do not undergo dye leaching.

Incubation with **PP**_**Rb**_ resulted in rapid cellular uptake (1 h) whilst remaining non-toxic up to 70 µM. Crucially, no localisation within the nucleus was observed under the limits of detection. Initial investigations were undertaken into the uptake mechanism and efficacy as a PP MP model. Increased mean lysosomal fluorescence intensity following **PP**_**Rb**_ treatment suggests an endosomal uptake mechanism with significant clearance implied over a 48 h period. Cellular localisation of **PP**_**Rb**_ is directed by the parent alcohol-functionalised copolymer, as evidenced by competition studies, indicating that **PP**_**Rb**_ is a valuable model for **PP** cell interaction_._ Optimistically, accounting for the constraints of our investigation, **PP**_**Rb**_ acts as an inert orthogonal species within the cell. However, it is important to investigate this further, especially by probing the polymer species as a vector for harmful environmental substances.

We believe this method for the synthesis and application of covalently bound dye moieties can elucidate the degree to which concern can be levied against the developing environmental plastic pollution. More broadly, this blueprint can enable a precise and confident interlaboratory analysis of polymer-cell interactions through the targeted synthesis of dye-modified polymers of interest. Additionally, this method offers many investigative opportunities, be it an *in vitro* cell line or *in vivo* studies of more complex organisms, including their ecological surroundings.

## Supplementary Material

**Supporting Information.**

The following files are available free of charge.

Electronic supporting information (PDF containing the experimental and data analysis methods, supporting polymer characterization, and images and data pertaining to the cell studies)

SI
