# Supplementary material for: Fluorescently Labelled Polypropylene as a Model Microplastic for Cellular Imaging
Source: Polym Chem. Author manuscript; Available in PMC 2026 Aug 20. (PMC7619382; doi:10.1039/d6py00089d)
Supplement: Supplementary file 1 [file EMS217508-supplement-SI.pdf]

Electronic Supplementary Information

Illuminating microplastics in cells

Alexander Evans‡, Ríona M. Devereux‡, Zoë R. Turner, Angela J. Russell,* and Dermot O'Hare*

Chemistry Research Laboratory, Department of Chemistry, University of Oxford, 12 Mansfield Road, OX1 3TA Oxford, UK.
E-mail: dermot.ohare@chem.ox.ac.uk

Contents

1. **General details and instrumentation** 2
2. **Synthesis and characterisation.** 5
3. **Cellular studies** 12
4. **PP_{Rb} hydrolysis control experiment.** 15
5. **References** 15

1. General details and instrumentation

General procedures. Dichloromethane (DCM) were dried using an MBraun SPS 800 solvent purification system, stored over activated 3 Å molecular sieves, and degassed under partial vacuum before use. Cryomilling of samples was conducted by submerging the polymer in liquid N_2 in a mortar, then applying a force using a pestle.

Commercially supplied materials. *N*,*N′*-Dicyclohexylcarbodiimide (DCC), 4-(Dimethylamino)pyridine (DMAP), Rhodamine B (Rb), 1,1,2,2-tetrachloroethane-d_2, chloroform, toluene were used as received from Sigma Aldrich.

Solution NMR spectroscopy. NMR spectra were recorded on a Bruker NEO 600 (14.1 T, 600.4 MHz) with a broadband helium cryoprobe. Spectra were recorded at 393 K and referenced internally to the residual *protio* solvent resonance. Chemical shifts, δ, are reported in parts per million (ppm) relative to tetramethylsilane (δ = 0 ppm). Convection compensated diffusion experiment was carried out using Bruker pulse program “dstebpgp3s” on a Bruker AVD 500 MHz, $C_2D_2Cl_4$, 393 K.

Gel permeation chromatography. Gel permeation chromatography (GPC) was performed by Ms Liv Thobru, Ms Sara Rund Herum, and Ms Rita Jenssen (Norner AS, Norway) on a high temperature gel permeation chromatograph with an IR5 infrared detector (GPC-IR5). Samples were prepared by dissolution in 1,2,4-trichlorobenzene (TCB) containing 300 ppm of 3,5-di-*tert*-buty-4-hydroxytoluene (BHT) at 160 °C for 90 minutes and then filtered with a 10 µm SS filter before being passed through the GPC column. The samples were run under a flow rate of 0.5 mL min^{-1} using TCB containing 300 ppm of BHT as mobile phase with 1 mg mL^{-1} BHT added as a flow rate marker. The GPC column and detector temperature were set at 145 and 160 °C respectively.

Differential scanning calorimetry. Differential scanning calorimetry was performed on a Perkin Elmer DSC 4000 System, unless otherwise stated, within a temperature range of 30-200 °C at a rate of 5 K min^{-1}. Polymer samples were sealed in 50 µL aluminium crucibles. An empty crucible was used as a reference, and the DSC was calibrated using indium and zinc.

Thermogravimetry. Thermogravimetric analysis was performed on a Perkin Elmer TGA 8000 thermogravimetric analyser within a temperature range of 50-800 °C. Polymer samples were loaded into pre-weighed ceramic pans and heated at a rate of 20 K min^{-1} unless otherwise stated. A purge gas of dry nitrogen was used.

Scanning Electron Microscopy. Scanning electron microscopy images were taken by adding samples as a solid powder to a carbon spot and placed on 12.5 mm aluminium stub before examining in a Jeol JSM6010. All samples were coated with platinum using a Quorum SC7620 Sputter Coater for 120 s before imaging. Accelerating voltages ranging from 5–15 kV were used.

Fourier-Transform Infrared Spectroscopy. Fourier-Transform Infrared Spectroscopy spectra were collected on a Bruker VERTEX 80 FT-IR spectrometer fitted with a DuraSamplIR Diamond ATR. Before the sample scans, 128 scans were taken as a background over the range 4000-400 cm^{-1}. Transmittance was recorded at a resolution of 4 cm^{-1} over the range 4000-400 cm^{-1} for 128 scans.

Ultraviolet–visible Spectroscopy. Optical measurement were conducted using UV-Vis-NIR spectrophotometer (PerkinElmer lambda 1050+). UV-Vis adsorption were measured by using a 150 mm diameter integrating sphere and 1 cm quartz cuvettes.

Dynamic Light Scattering. Hydrodynamic diameters (D_h) and size distributions were determined by dynamic light scattering on a MALVERN Zetasizer Nano ZS operating at 90 °C with a 4 mW He–Ne 633 nm laser module. Measurements were made at a detection angle of 173° (back scattering). Measurements were repeated three times with automatic attenuation selection and measurement position. The results were analysed using Malvern DTS 6.20 software. PDI values were calculated using

eqn (1), where σ is the standard deviation and *d* is the diameter both obtained from the number, intensity, and volume distribution where relevant: (1) $PDI = \sigma^2/d^2$

Fluorescence Spectroscopy. Fluorescence intensity was monitored using a Shimadzu RF-6000 at an excitation wavelength of 558 nm and collection of emission at 581 nm based on using a quartz cuvette with a 1 cm path length.

Cell Culture. HEK293T cells (CRL-3216, ATCC, Manassas, VA, USA) were grown in Dulbecco's modified Eagle's medium (DMEM) containing high glucose (4.5 g/L, Sigma), 2 mM *L*-glutamine (Sigma), 10% heat-inactivated fetal bovine serum (FBS) (Sigma), 100 U/mL penicillin-streptomycin (Sigma) at 5% CO_2 and 37 °C.

AlamarBlue® cell viability assay. HEK293T cells were seeded in 96-well plates and allowed to adhere and grow for approximately 24 h in maintenance media (100 µL) at 37 °C and 5% CO_2. The cells were then treated with varying concentrations of $\mathbf{PP_{Rb}}$ for 24 h (final DMSO concentration <0.3%). $\mathbf{PP_{Rb}}$ concentrations are calculated based on the theoretical molar mass ($M_{n,\ th}$) of $\mathbf{PP_{Rb}}$ predicted ca. 14 700 g mol^{-1}, based on parent copolymer experimental molar mass ($M_{n,\ SEC}$ = 13 800 g mol^{-1}) with quantitative conversion of the 1.87 mol% Br moiety. AlamarBlue® reagent (Thermo Fisher Scientific) (10 µL) was added directly into the cell culture media, and the cells were incubated in the dark for a further 4 h at 37 °C and 5% CO_2. The fluorescence (540-570 nm) and absorbance (570 and 600 nm) were then measured using a FLUOstar Omega plate reader.

Confocal imaging of Rb and $\mathbf{PP_{Rb}}$. HEK293T cells were seeded onto fibronectin-treated coverslips and allowed to adhere for 24 h. The cells were then treated with 35 µM **Rb** or $\mathbf{PP_{Rb}}$ for various durations (1 or 24 h), after which the cells were washed with DPBS, fixed with 4% PFA for 10 min at RT, and washed thrice with DPBS. Coverslips were mounted on slides using Fluoromount-G™, with DAPI (Invitrogen) and allowed to set overnight at 4 °C. The cells were scanned using a Leica TCS SP5 confocal microscope (555: λ_{ex} = 545/ λ_{em} = 566 nm, DAPI: λ_{ex} = 358/ λ_{em} = 461 nm) and acquired images were processed using Fiji software.

***In vitro* competition of $\mathbf{PP_{Rb}}$ with $\mathbf{PP_{EAE}}$.** HEK293T cells were seeded onto fibronectin-treated coverslips and allowed to adhere for 24 h. The cells were then treated with 35µM $\mathbf{PP_{Rb}}$ and 35 µM $\mathbf{PP_{EAE}}$ for various durations, after which the cells were washed with DPBS, fixed with 4% PFA for 10 min at RT, and washed thrice with DPBS. Coverslips were mounted on slides using Fluoromount-G™, with DAPI (Invitrogen) and allowed to set overnight at 4 °C. The cells were scanned using a Leica TCS SP5 confocal microscope (555: λ_{ex} = 545/ λ_{em} = 566 nm, DAPI: λ_{ex} = 358/ λ_{em} = 461 nm) and acquired images were processed using the Colocalisation Colourmap plugin for Fiji software.

***In vitro* staining of lysosomes.** HEK293T cells were seeded onto fibronectin-treated coverslips and allowed to adhere for 24 h. The cells were then treated with 35 µM **Rb** or $\mathbf{PP_{Rb}}$ for various durations, after which the cells were washed with DPBS and then incubated with pre-warmed DMEM with 50 nM of Lysotracker® Green DND-26 (Thermo Fisher Scientific) for 30 min at 37 °C. The cells were washed with DPBS, fixed with 4% PFA for 10 min at RT, and washed thrice with DPBS. Coverslips were mounted on slides using Fluoromount-G™, with DAPI (Invitrogen) and allowed to set overnight at 4 °C. The cells were scanned using a Leica TCS SP5 confocal microscope (LysoTracker: λ_{ex} = 504/ λ_{em} = 511 nm, DAPI: λ_{ex} = 358/ λ_{em} = 461 nm) and acquired images were processed using Fiji software.

***In vitro* staining of polymer colocalisation with lysosomes.** HEK293T cells were seeded onto fibronectin-treated coverslips and allowed to adhere for 24 h. The cells were then treated with 35 µM **Rb**, $\mathbf{PP_{Rb}}$ or $\mathbf{^{C}PP_{Rb}}$ for various durations, after which the cells were washed with DPBS and then incubated with pre-warmed DMEM with 50 nM of Lysotracker® Green DND-26 (Thermo Fisher Scientific) for 30 min at 37 °C. The cells were washed with DPBS, and immediately scanned using a

Leica TCS SP5 confocal microscope (LysoTracker: $\lambda_{ex} = 504/ \ \lambda_{em} = 511$ nm, 555: $\lambda_{ex} = 545/ \ \lambda_{em} = 566$ nm, DAPI: $\lambda_{ex} = 358/ \ \lambda_{em} = 461$ nm) and acquired images were processed using Fiji software.

Statistical analysis. All experimental samples were run in triplicate with at least three biological repeats. Differences between samples were considered significant if $p < 0.05$ as determined using either a Student's T-test or a one-way Analysis of Variance (ANOVA). All graphs were created using OriginPro 2024b.

2. Synthesis and characterisation.

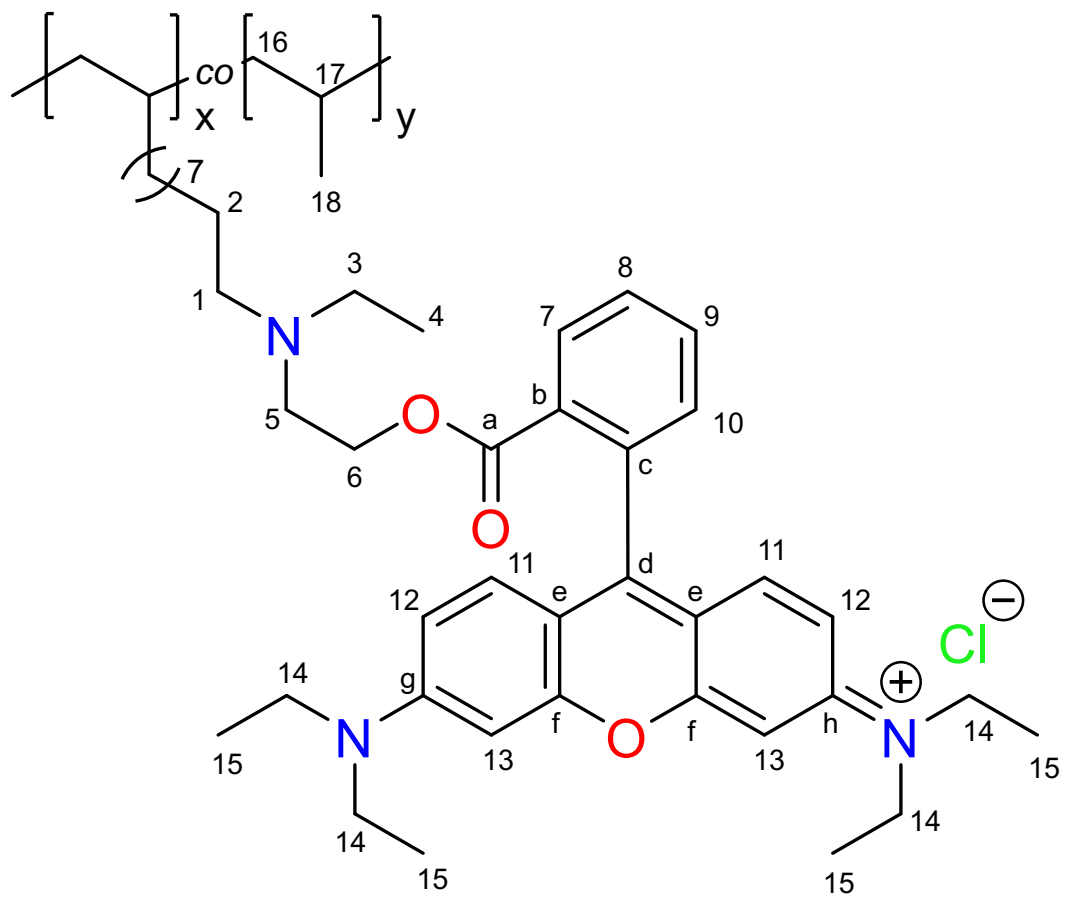

Synthesis of Rhodamine B modified PP_{EAE}, PP_{Rb}. PP_{EAE} (0.5 g, 1.0 eq, 1.87 mol% [R-OH]), Rhodamine B (2.0 eq., Rb)), DCC (2.0 eq.), and DMAP (0.3 eq.) were dissolved in anhydrous DCM (50 ml) in an ampoule, sparged under N_2 and stirred for 30 min prior to sealing and heating to 80 °C for 72 h. Polymer was precipitated in methanol (2 wt%) before being collected by filtration, washed with methanol (20 mL), then acetone (2 x 20 mL), and dried *in vacuo* at 40 °C to a constant mass. Polymer was then purified by Soxhlet extraction (H_2O), ensuring polymer was trapped at the base of the thimble, until no residual Rb was observed in the solvent by thin layer chromatorgaphy (DCM). Polymer was then washed with acetone and dried *in vacuo* at 40 °C to a constant mass.**PP_{Rb}** (1.87 mol%). **^{1}H NMR (600 MHz, $C_2D_2Cl_4$, 393 K)**: δ 7.90 (1H, H^7), 7.48 (2H, $H^{8.9}$), 7.08 (1H, H^{10}), 6.56 (2H, H^{12}), 6.53 (2H, H^{13}), 6.40 (2H, H^{11}), 4.00 (6H, br s, H^6), 3.58, 3.44 (8H. q, $^3J_{HH}$ = 7.0 Hz, H^{14}), 3.25-3.00 (18H, H^3, H^5, and H^1), 2.02-1.62 (217H, br m, H^{15}, H^2, H^{17}), 1.55-1.06 (432H, br m, H^b, $H^{16\ (anti)}$, and H^4), 1.05-0.84 ppm (725H, br m, H^{18} and $H^{16\ (syn)}$).**^{13}C{^{1}H} NMR (151 MHz, $C_2D_2Cl_4$, 393 K)**: δ 201.02 (C^b), 190.84 (C^b), 158.16 (C^b), 156.16 (C^b), 156.24 (C^b), 132.93 (C^b), 131.79 (C^b), 130.52 (C^b), 130.19 (C^b), 130.03 (C^b), 123.94 (C^b), 120.54 (C^b), 116.68 (C^b), 116.47 (C^b), 116.27 (C^b), 114.12 (C^b), 111.24 (C^b), 100.31 (C^b), 99.88 (C^b), 96.97 (C^b), 65.50 (C^{14}), 58.73 (C^5), 56.79 (C^6), 54.17 (C^1), 49.45 (C^3), 46.22 (C^{17}), 28.58 (C^{16}), 23.47 (C^2), 21.66 (C^{18}), 8.81 (C^4).**FTIR**: v (cm^{-1}) = 2950, 2867, and 1456 (v (C-H, CH_3)), 2917, 2838, and 1377 (v (C-H, CH_2)), 1619 (v (C=O)), and 808 (v (C=C_{Ar})). **Fluorescence**: λ_{Ex} = 558 nm and λ_{Em} = 581 nm (0.5 mg mL^{-1}, chloroform). **UV-Vis**: λ_{Ex} = 558 nm (0.5 mg mL^{-1}, chloroform). [a]Nitrogen adjacent ^{1}H NMR peaks appear broad. This observation is potentially explained by an increase in the relaxation rate caused by quadrupolar Nitrogen.[1, 2] [b]Peaks corresponding to unassigned 20 carbons on Rb (7-13 and a-g). Due to the low levels of dye incorporation it was difficult to assign or integrate corresponding Rb moiety and comonomer -CH_2- peaks in the ^{13}C NMR spectrum; complimentary use of COSY and HSQC spectra afforded partial assignment.

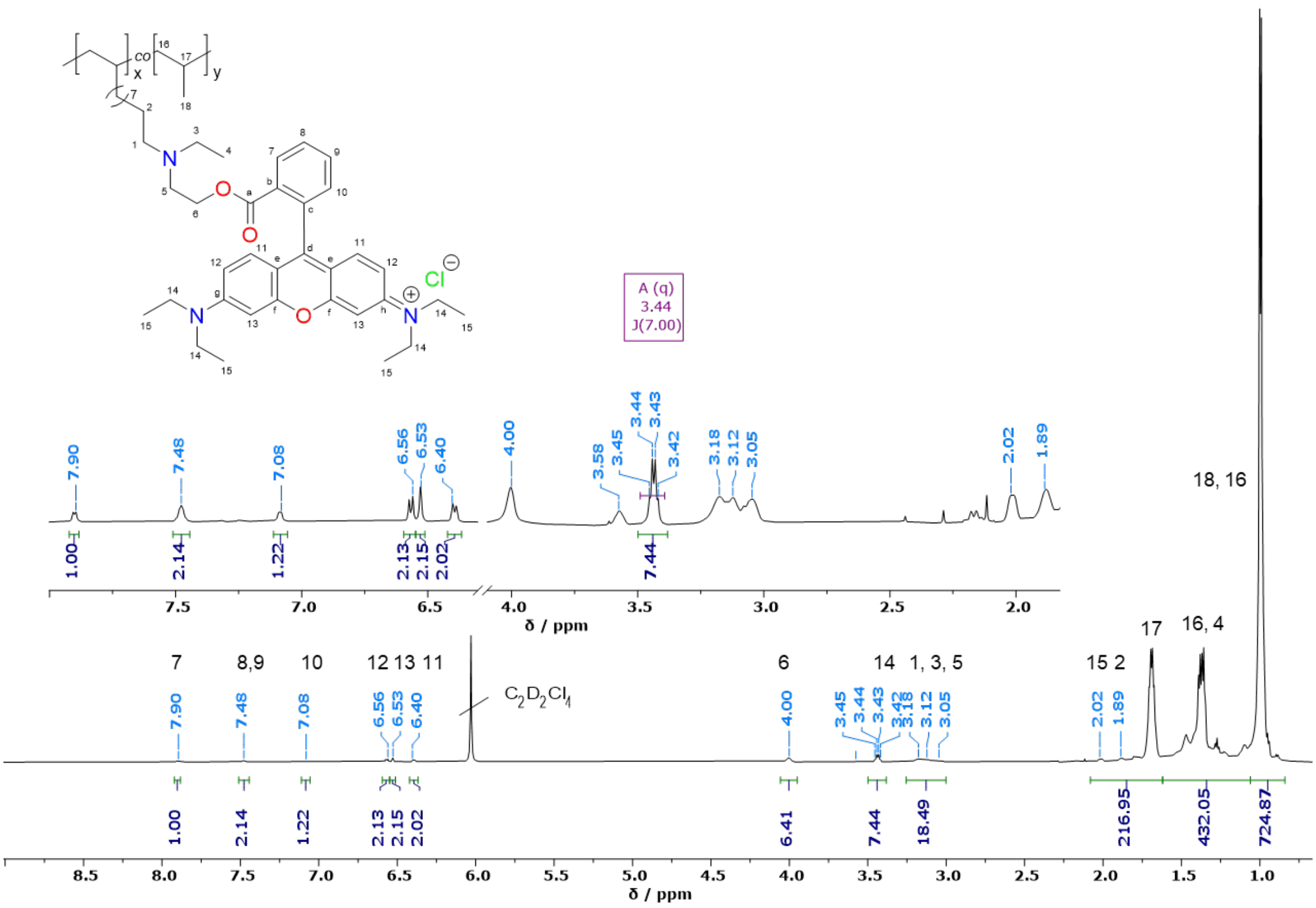

Figure S1 PP$_{Rb}$ ^{1}H NMR (600 MHz, $C_2D_2Cl_4$, 393 K). Insert expanded region corresponding to polymer linker and dye moiety.

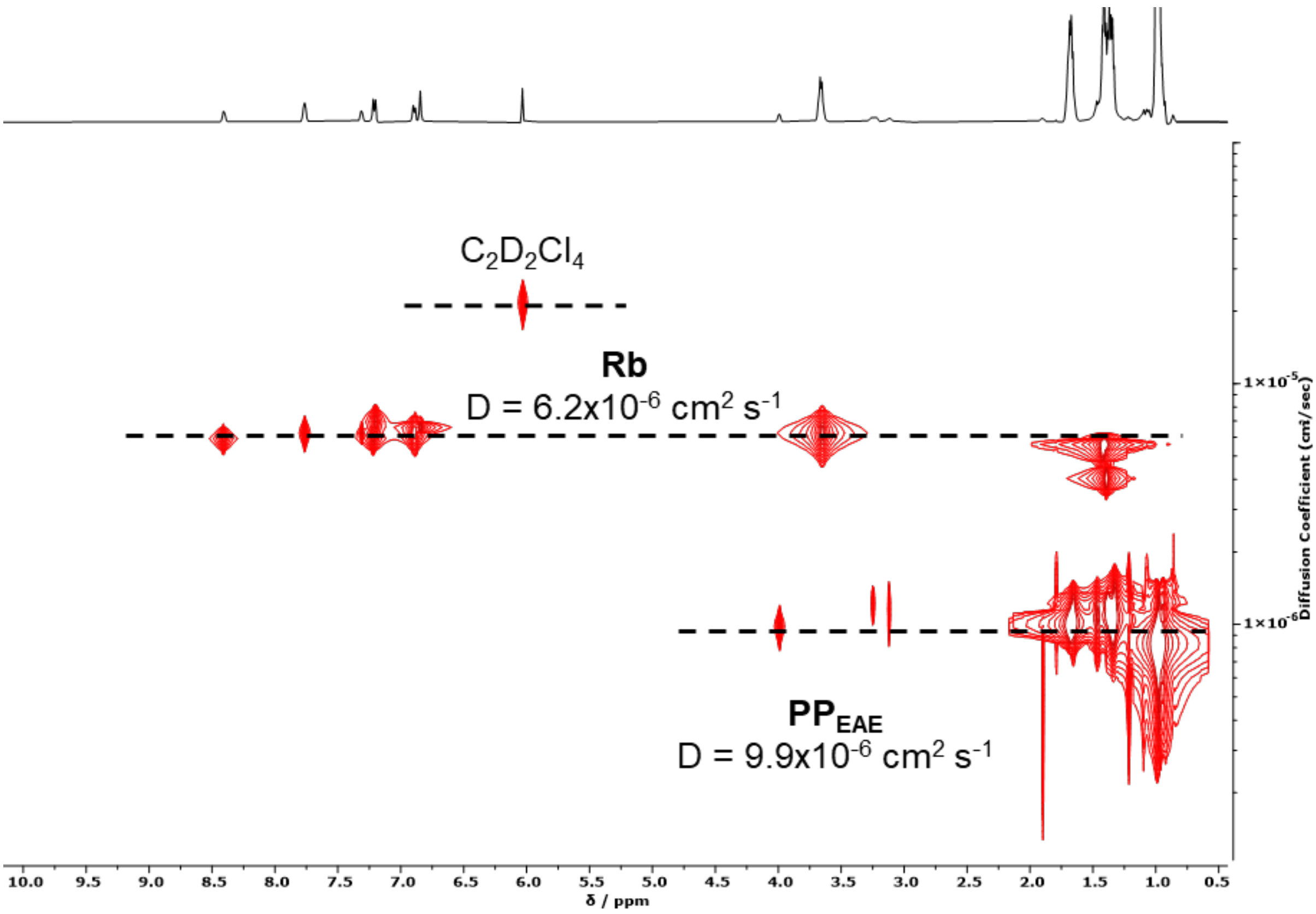

Figure S2 Convection compensated diffusion experiment using Bruker pulse program "*dstebpgp3s*" (500 MHz, $C_2D_2Cl_4$, 393 K). DOSY ^{1}H NMR spectra of parent polymer **PP$_{EAE}$** + **Rb**.

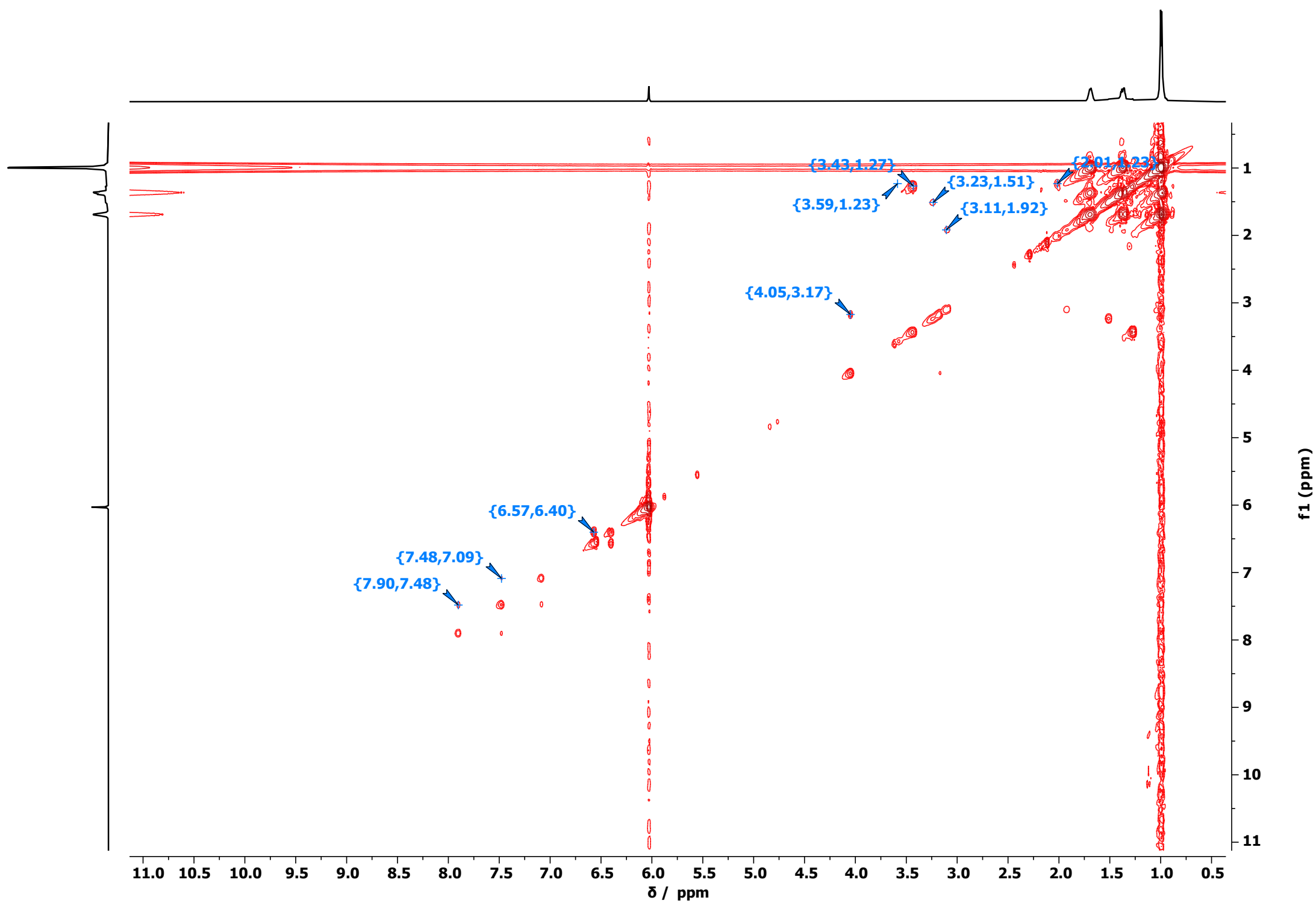

Figure S3 COSY ^{1}H-^{1}H NMR spectrum

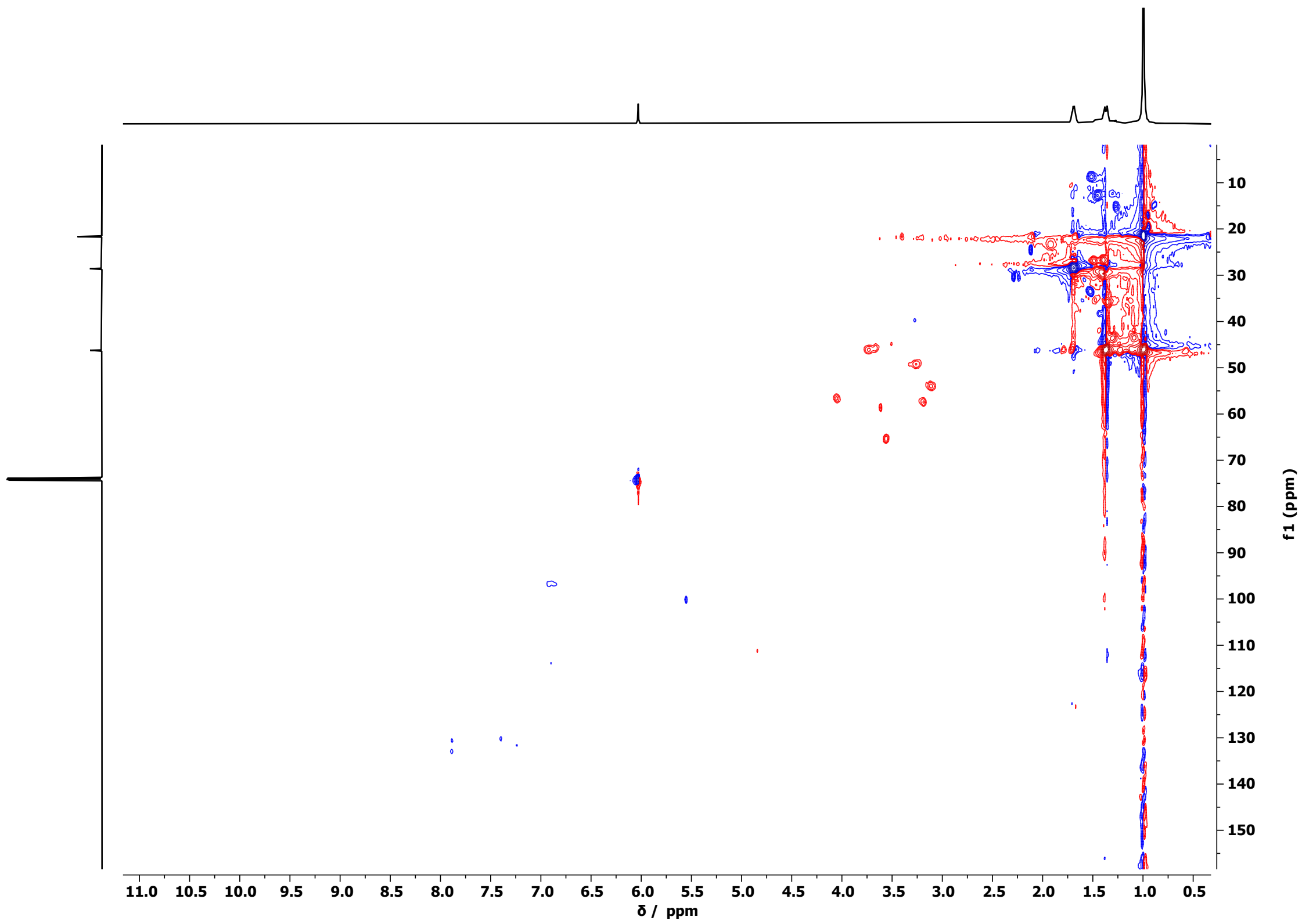

Figure S4 HSQC ^{1}H-^{13}C NMR spectrum

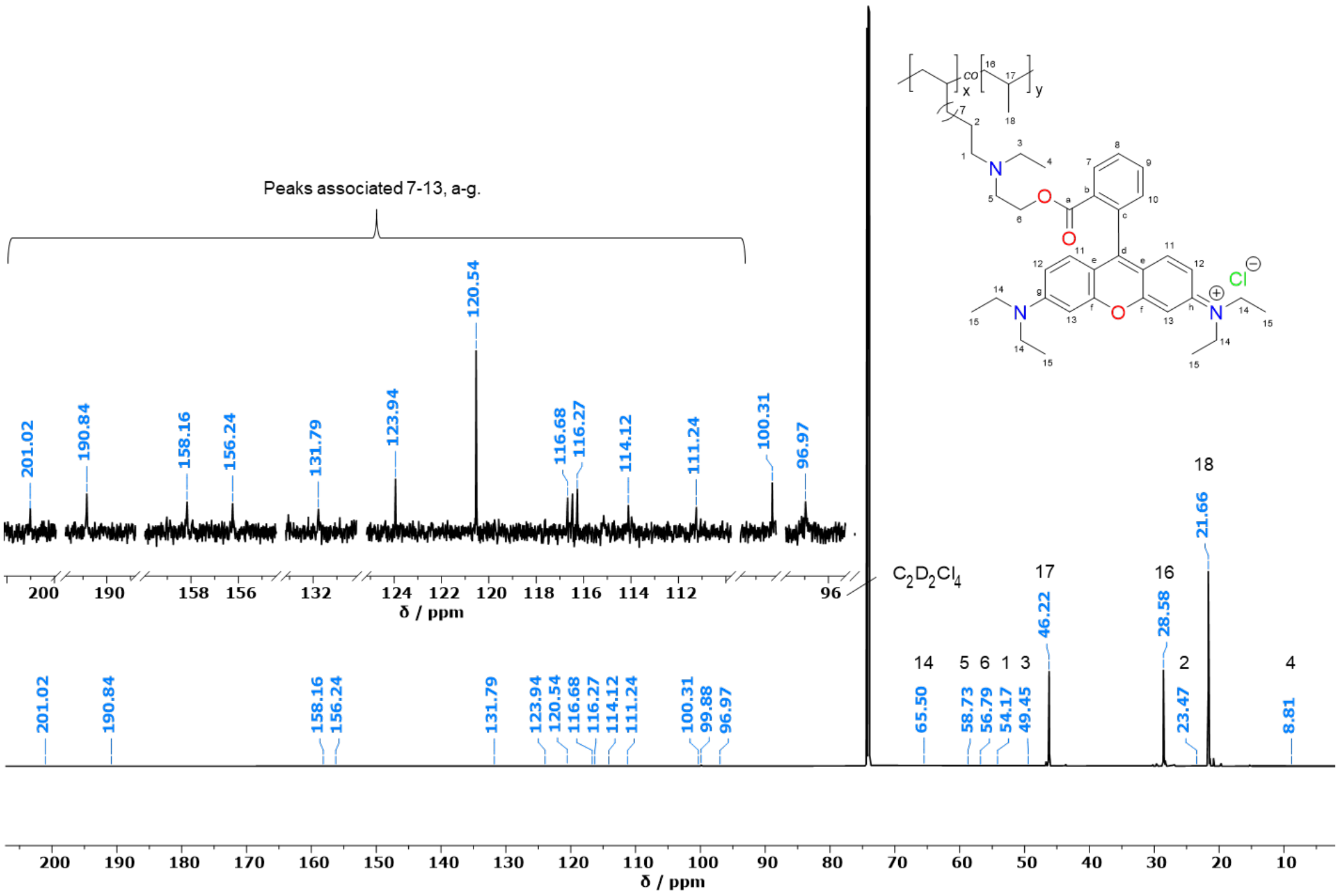

Figure S5 PP$_{Rb}$ $^{13}C\{^1H\}$ NMR (151 MHz, $C_2D_2Cl_4$, 393 K), full spectrum.

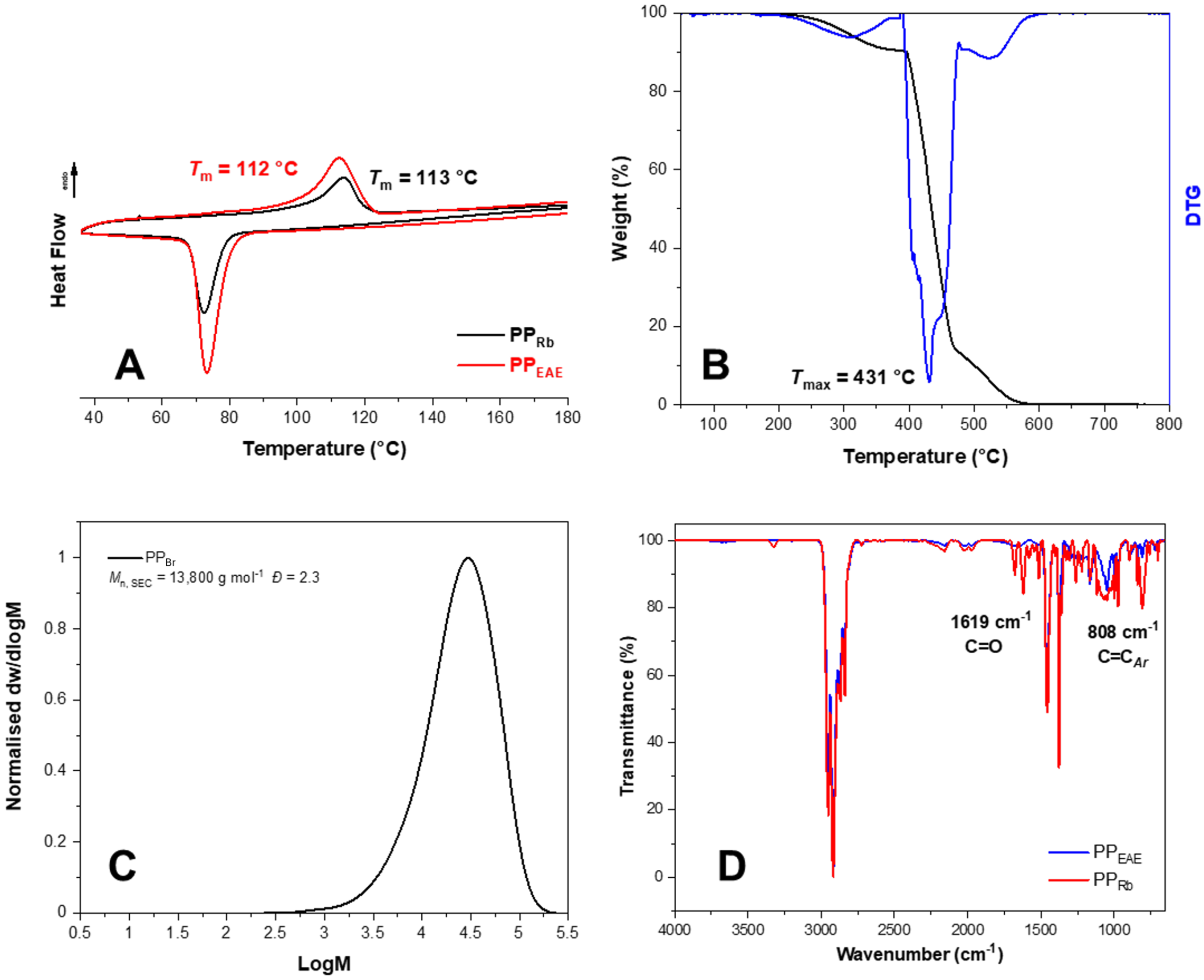

Figure S6 (**A**) DSC trace of **PP$_{Rb}$** and **PP$_{EAE}$**, temeperature scan rate 5 °C min^{-1}; observed decrease in crystallinity upon modification (**PP$_{EAE}$** = 35%, **PP$_{Rb}$** = 29%) (**B**) TGA thermograph of PP$_{Rb}$ in a N_2 atmosphere, heating rate 20 °C min^{-1}. (**C**) **PP$_{Br}$** parent copolymer used to calculate **PP$_{Rb}$** $M_{n,\,th}$ = (13,800–(1.87×79.9)+(1.87×89.14)+(1.87×(479.02-2)) (g mol^{-1}))). (**D**) FT-IR of **PP$_{Rb}$** and **PP$_{EAE}$**.

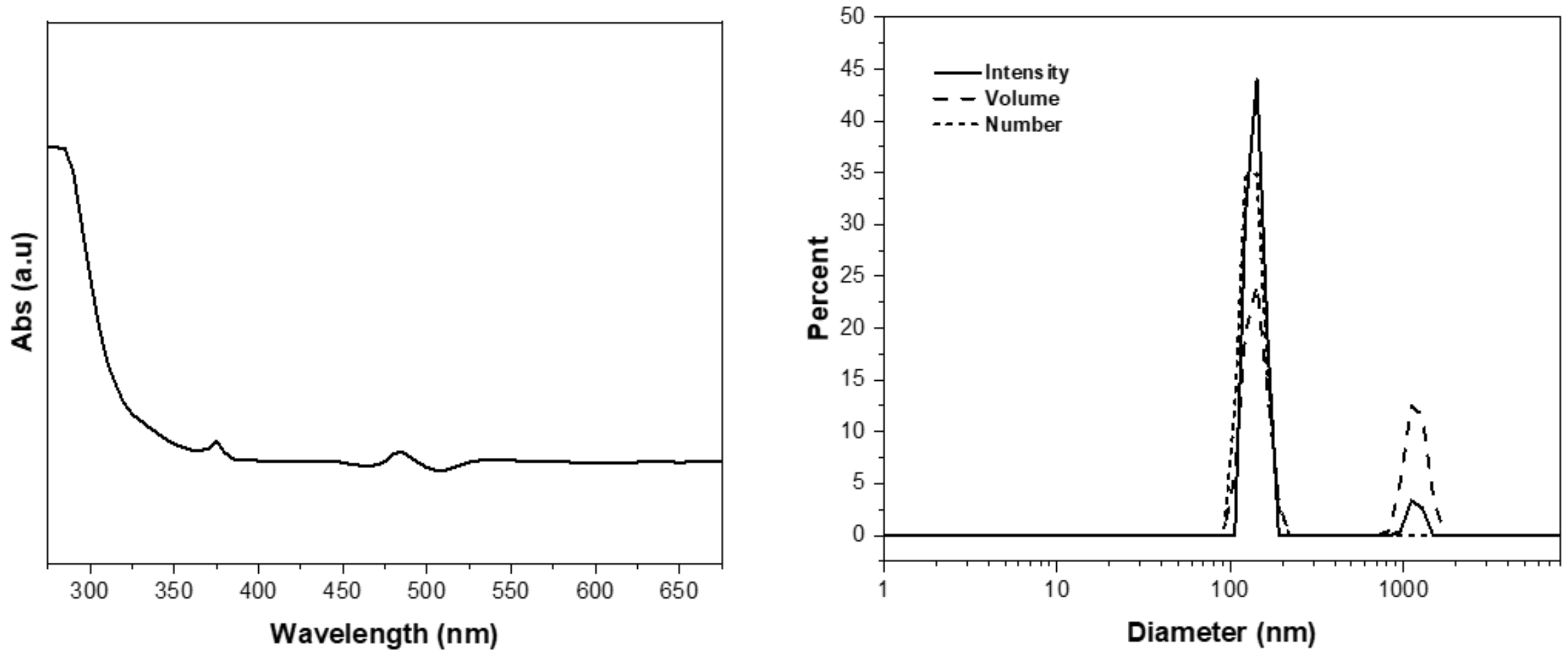

Figure S7 **PP$_{EAE}$** UV-Vis and DLS (1 mg ml^{-1}, toluene)

Table S1 Particle size data of **PP$_{Rb}$** and **PP$_{EAE}$** by dynamic light scattering (1 mg ml^{-1}, toluene)

**Polymer**	**%**	D_h	**PDi**
**PP$_{Rb}$**	Intensity	566.2	0.029
	Volume	588.1	0.039
	Number	547.6	0.039
**PP$_{EAE}$**	Intensity	139.9	0.011
		1,170	0.007
	Volume	139.8	0.022
		1,182	0.018
	Number	135.3	0.021
		1,155	0.019

$$\mathbf{PDi} = \sigma^2/D_h^{\,2}$$

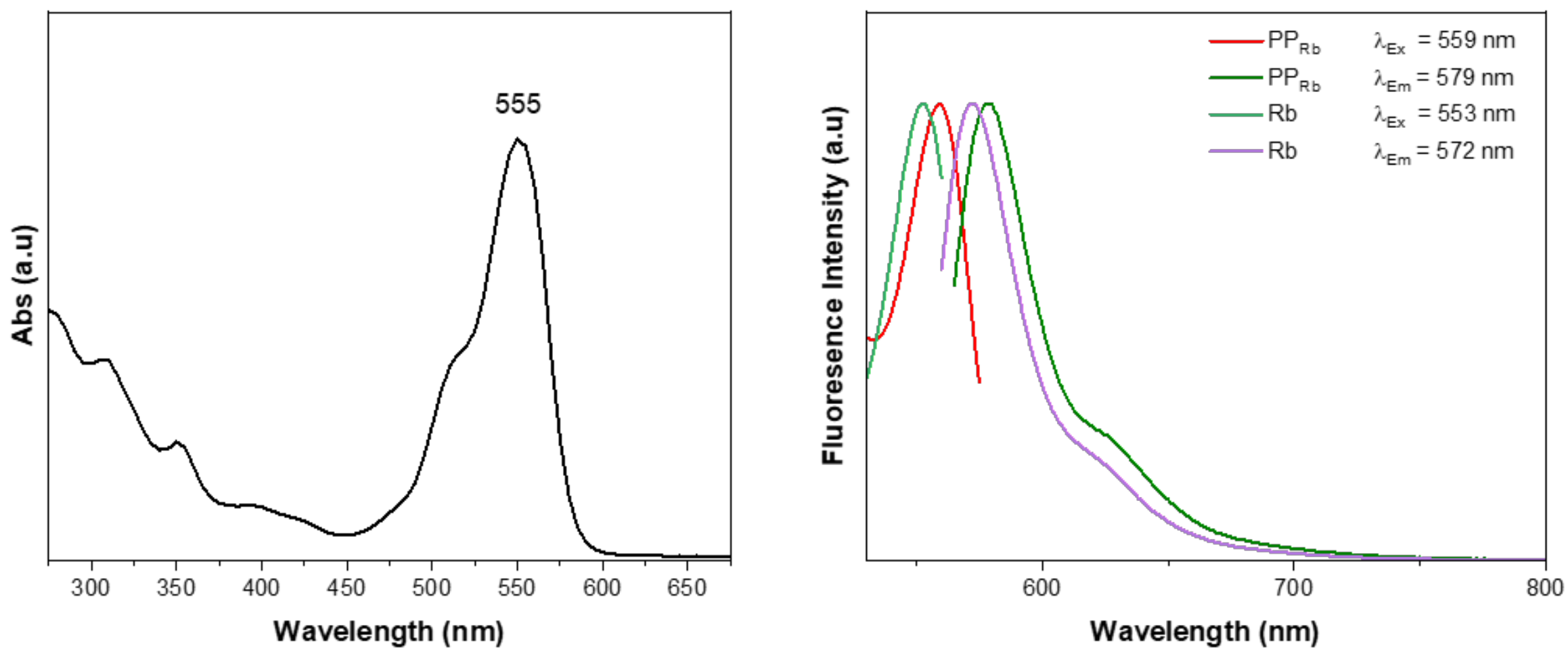

Figure S8 Rb (left) UV-Vis and (right) fluorescent spectra (0.522 nM, chloroform)

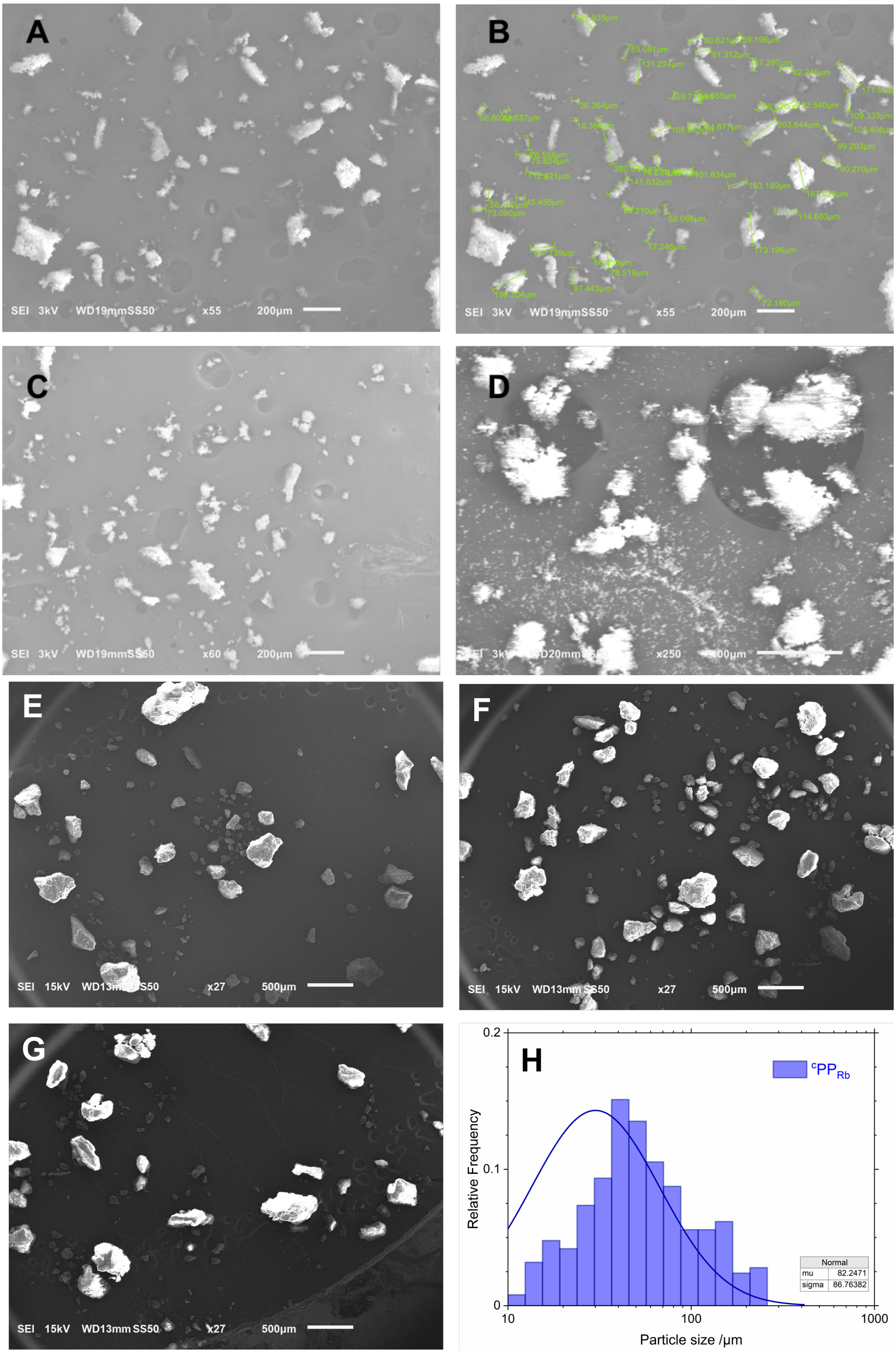

Figure S9 SEM images of **(A–D) PP_{Rb}** and **(E–G) $^{c}PP_{Rb}$** with accompanying particle size distribution of $^{c}PP_{Rb}$.

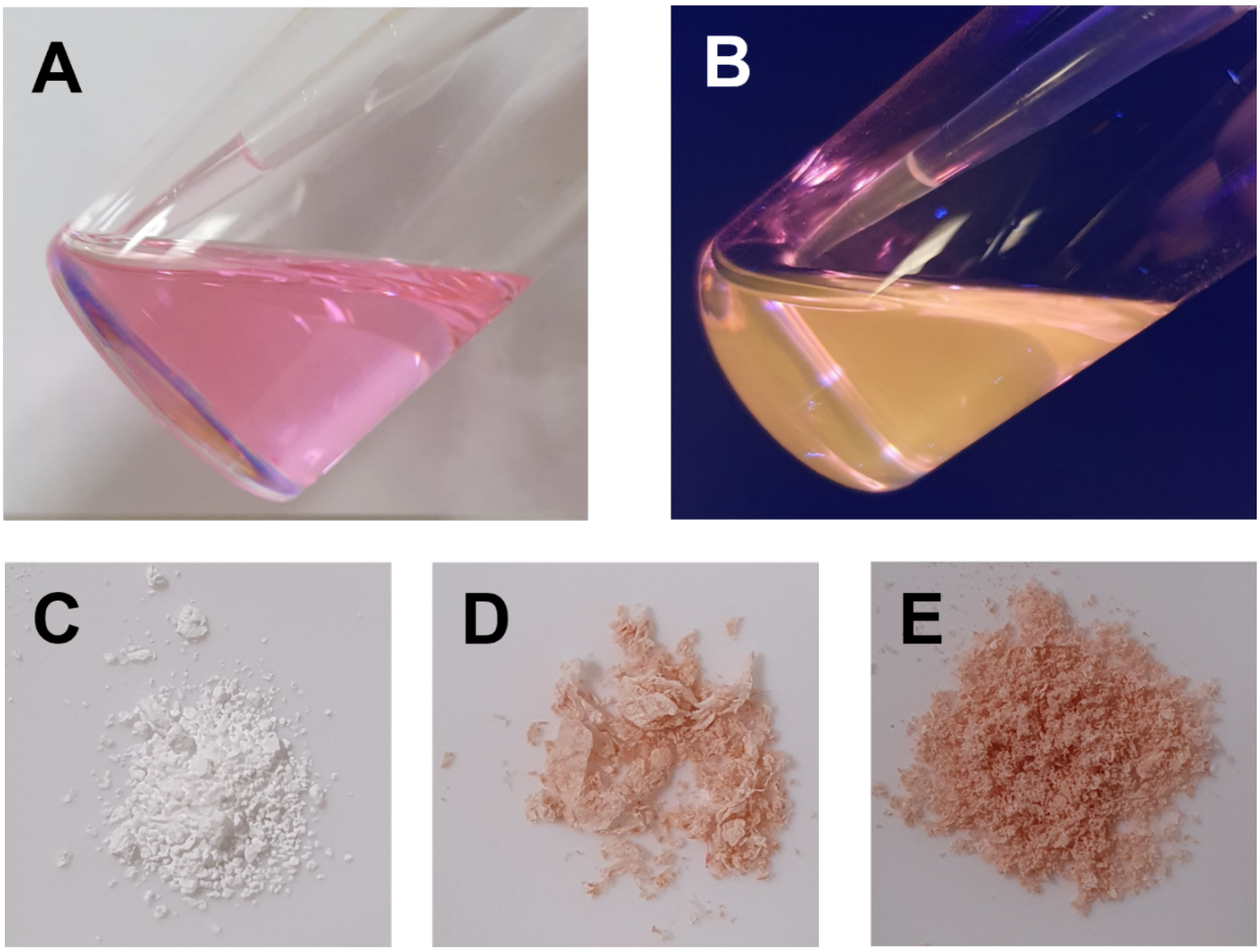

Figure S10 **PP_{Rb}** (0.5 mg mL^{-1}, chloroform) (**A**) visible light and (**B**) UV light. Photos of (**C**) **PP_{EAE}** (**D**) **PP_{Rb}** and (**E**) **$^{c}PP_{Rb}$**.

3. Cellular studies

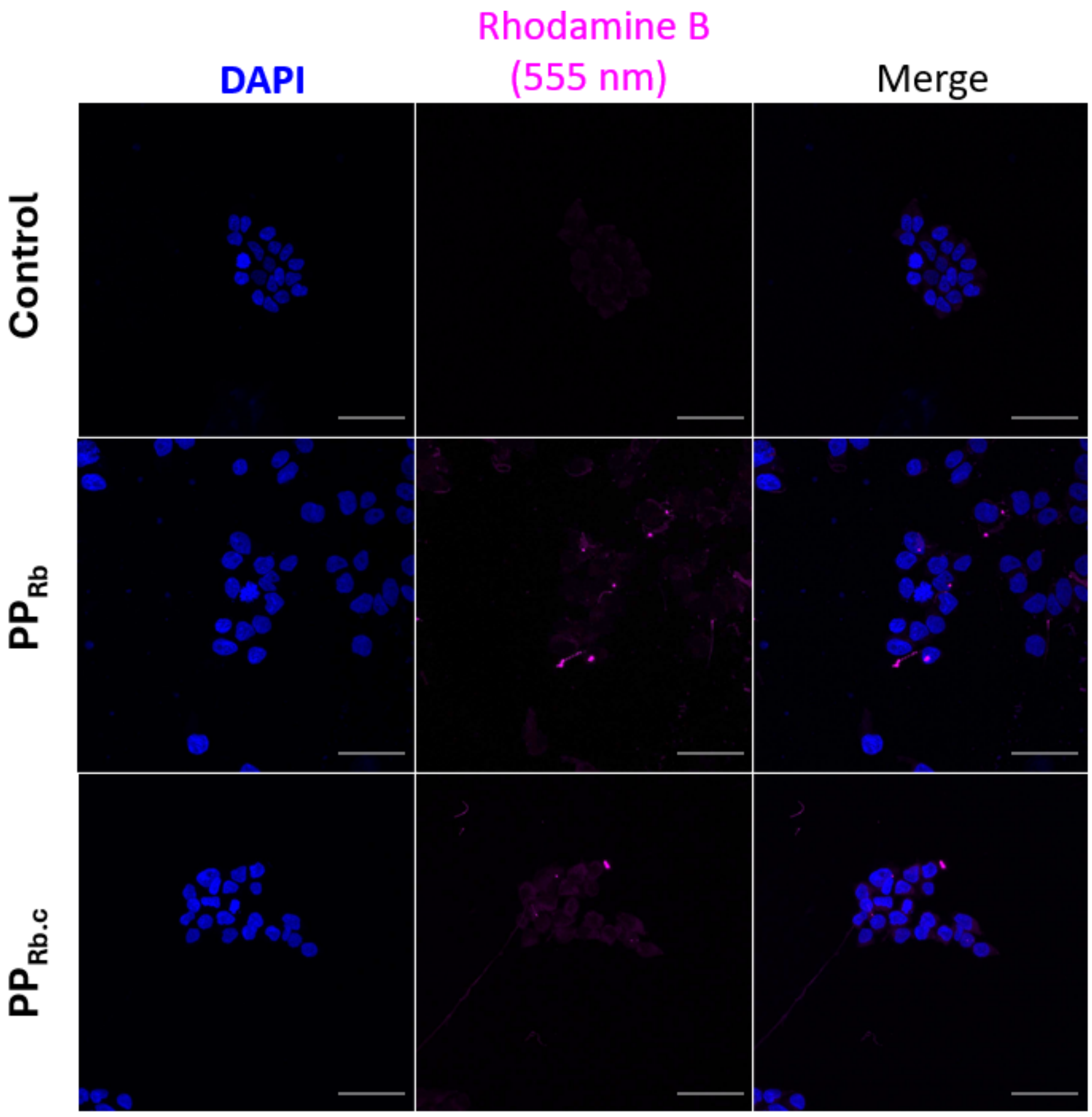

Figure S11 Comparable cellular localisation of both $\mathbf{PP_{Rb}}$ and $^{c}\mathbf{PP_{Rb}}$. Confocal microscopy images showing DAPI (nuclear stain), **Rb** fluorescence (555 nm), and merged channels for Control, $\mathbf{PP_{Rb}}$, and $^{c}\mathbf{PP_{Rb}}$ conditions after 24 h incubation. Scale bars 50 µm.

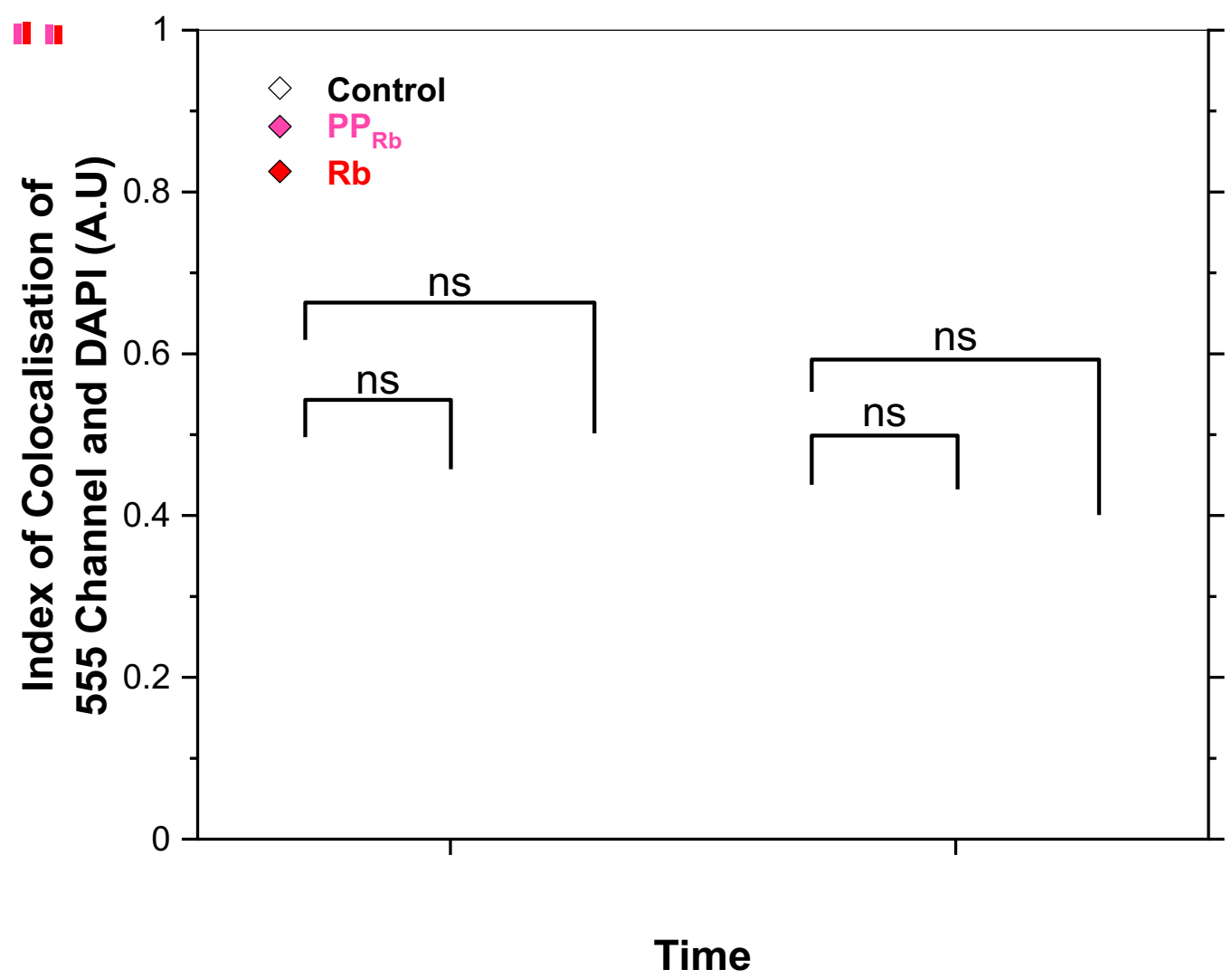

Figure S12 Analysis of Colocalisation between the 555 nm channel and DAPI for $\mathbf{PP_{Rb}}$ and **Rb**. The Index of Colocalisation between the 555 nm fluorescence signal and DAPI nuclear stain was statistically nonsignificant (ns) across all conditions (Control, $\mathbf{PP_{Rb}}$, and **Rb**) and time points (1 and 24 h).

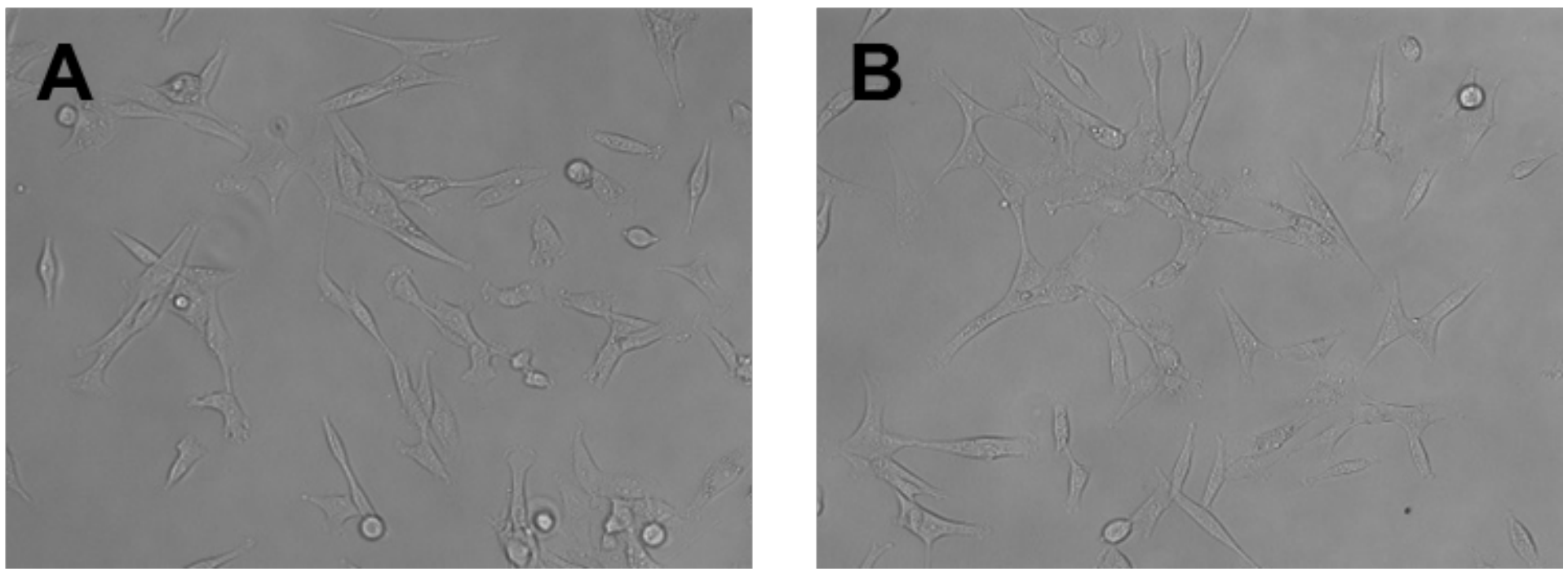

Figure S13 Representative bright-field microscopy images of HEK293 cells following 24 h exposure to 0 µM **PP$_{Rb}$** (**A**) and 35 µM **PP$_{Rb}$** (**B**), showing no observable signs of cell death or structural abnormalities between treated and untreated cells.

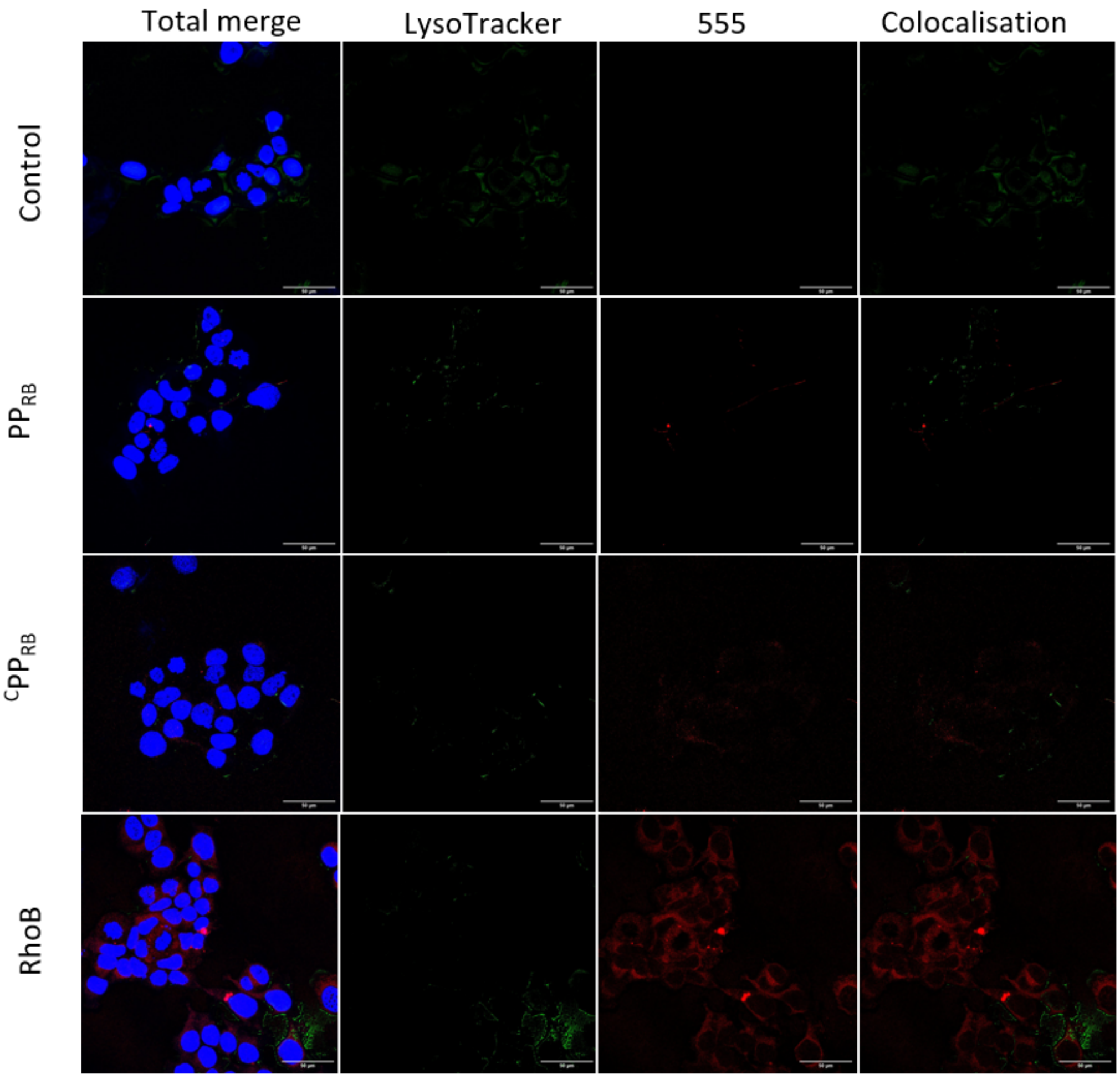

Figure S14. Quantification of colocalisation of **PP$_{Rb}$**, **^{c}PP$_{Rb}$**, and free **Rb** with lysosomes in HEK293T cells after 24 h. Confocal microscopy images showing lysosomal staining with LysoTracker (green), nuclear staining with DAPI (blue) and 555 channel (red) in control cells and cells treated with 35 µM of **PP$_{Rb}$**, **^{c}PP$_{Rb}$**, or free **Rb** for 24 h. Scale bars = 50 µm.

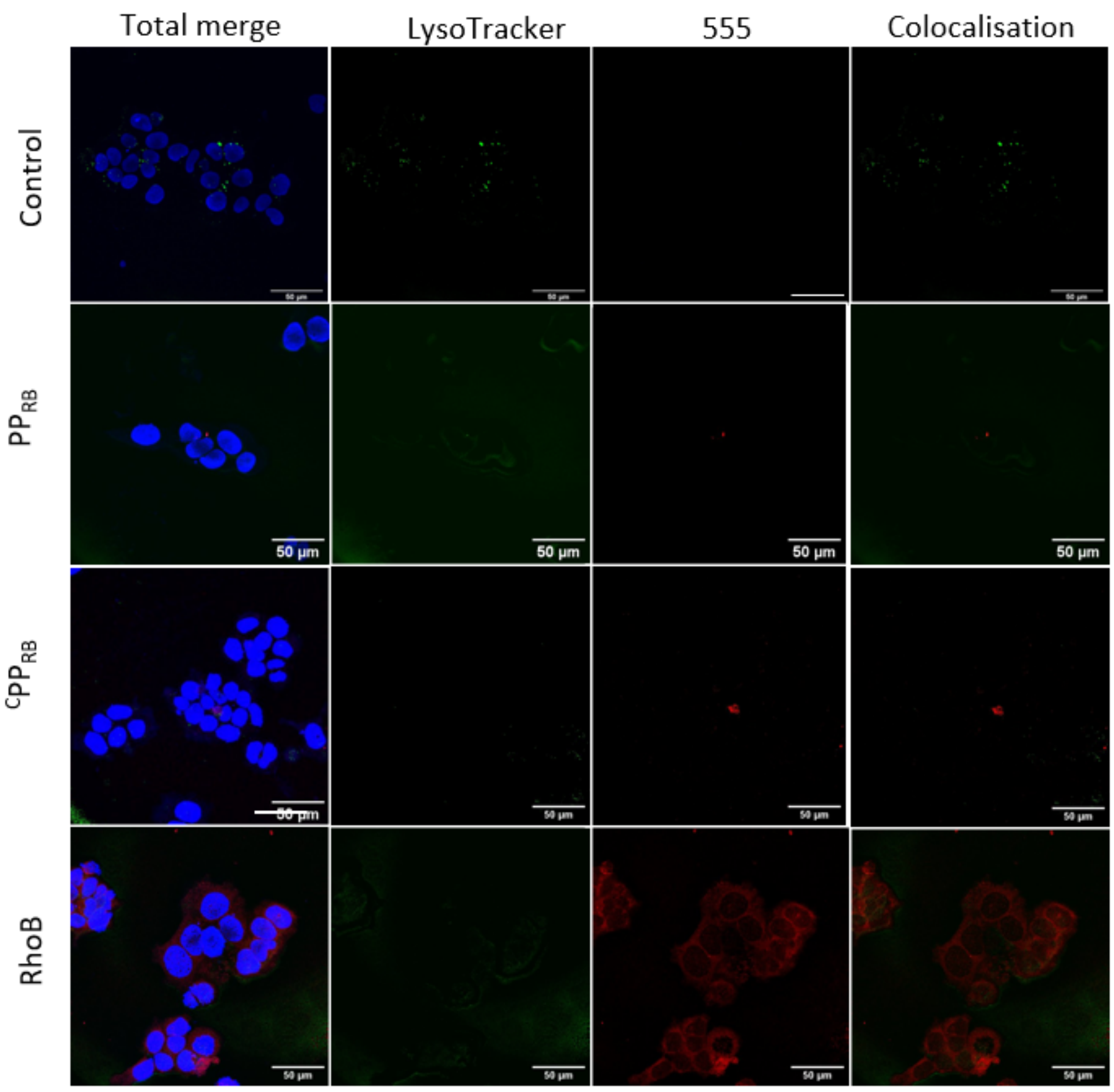

Figure S15. Quantification of colocalisation of **PP**$_{\mathbf{Rb}}$, ^{c}**PP**$_{\mathbf{Rb}}$, and free **Rb** with lysosomes in HEK293T cells after 48 h. Confocal microscopy images showing lysosomal staining with LysoTracker (green), nuclear staining with DAPI (blue) and 555 channel (red) in control cells and cells treated with 35 µM of **PP**$_{\mathbf{Rb}}$, ^{c}**PP**$_{\mathbf{Rb}}$, or free **Rb** for 48 h. Scale bars = 50 µm.

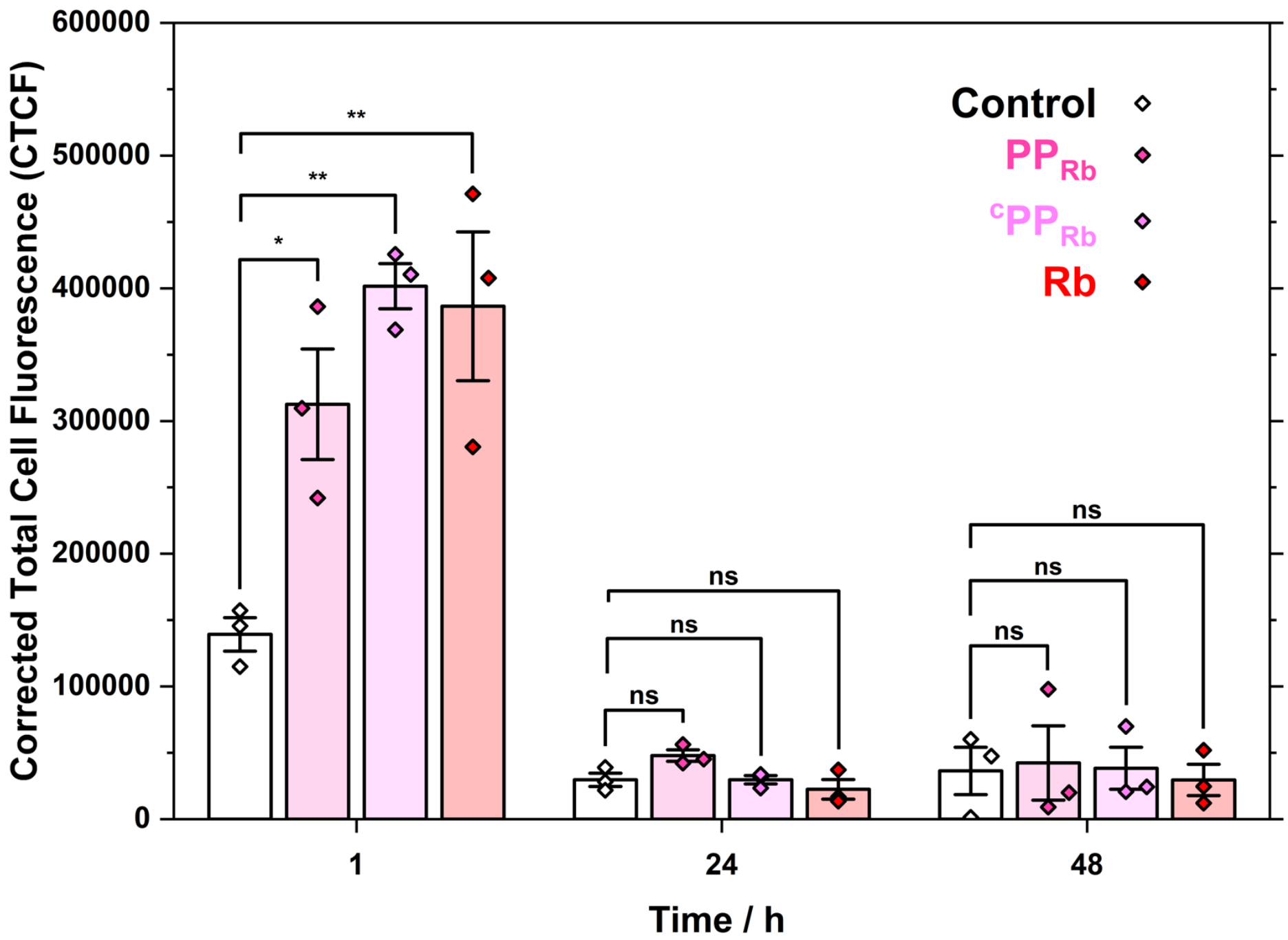

Figure S16 Quantification of lysosome formation in HEK293T cells following treatment with **PP**$_{\mathbf{Rb}}$, ^{c}**PP**$_{\mathbf{Rb}}$, and free **Rb**. Normalised LysoTracker Green fluorescence intensity (A.U.) was quantified over time (1, 24, and 48 h) for each treatment group (See Figures 4C, S14 and S15 for images). Data are represented as mean compared to control ± SEM (*n* = 3). Brown-Forsythe and Welch one-way ANOVA, non-parametric ns = $p > 0.05$, *$p \leq 0.05$and **$p < 0.01$ compared to control. Scale bars = 50 µm.

4. PP_{Rb} hydrolysis control experiment.

A physiological citrate-phosphate buffer solution mimicking the acidic lysosomal compartments at pH 4 was prepared according to reported literature.[3] The buffer was equilibrated and held at 38 °C for the duration of the experiment. A control dialysis experiment containing deionised water at pH 7 was likewise held at 38 °C for the duration of the experiment. $\mathbf{PP_{Rb}}$ (100 mg) was placed inside of pre-wetted, clipped 1 kDa MWCO dialysis tubing (SpectrPor) with the media agitated by a magnetic stirrer. Aliquots of dialysis media were removed periodically (t = 0, 1, 2, 3, 5, 6, and 7 days) and examined by fluorescence spectroscopy. Results are plotted in Figure S14A. Gradual increase fluorsence intensity were observed over the 7 days of the experiment. Fluorescence intensity at t = 7 days was similar for both pH 4 and pH 7 experiments suggesting the recorded fluorescence was not caused by the hydrolysis and escape of the dye moiety. ^{1}H NMR spectrum of the dialysis media after 7 days evidenced the presence of $\mathbf{PP_{Rb}}$ as shown by distinct peaks relating to the propylene backbone protons. Consequently it is proposed that as the polymer is inherently a distribution, it stands to reason that some $\mathbf{PP_{Rb}}$ is below the MWCO of the dialysis tubing. Furthermore, the polymer is not solvated in H_2O and consequently the hydrodynamic radius of $\mathbf{PP_{Rb}}$ will be different and may not behave as standard with respect to the MWCO of the dialysis tubing, enabling escape. However, assuming that all recorded fluorescence intensity is as a result of hydrolysis and escape of the dye moiety, a **Rb** calibration curve was plotted to calculate the maximum quantity of theoretically recorded free dye (Figure S14B). Using t = 3 days, the maximum experiment length undertaken, it correlates to 5.09×10^{-9} mol dm^{-3} of **Rb**. Given 100 mg of $\mathbf{PP_{Rb}}$, the maximum available dye based on a 1.87 mol% incorporation level and a M_n = 14 700 g mol^{-1}, [**Rb**] = 3.2×10^{-7} mol dm^{-3}, and a maximum percentage of hydrolysed $\mathbf{PP_{Rb}}$ = 1.6% after 3 days at pH 4 and 38 °C. This was deemed to not be statistically significant, especially when taken in conjunction with previously reported hydrolysis experiments of water soluble polymers containing the similarly esterified **Rb**[4] and the additional experimental observations described above.

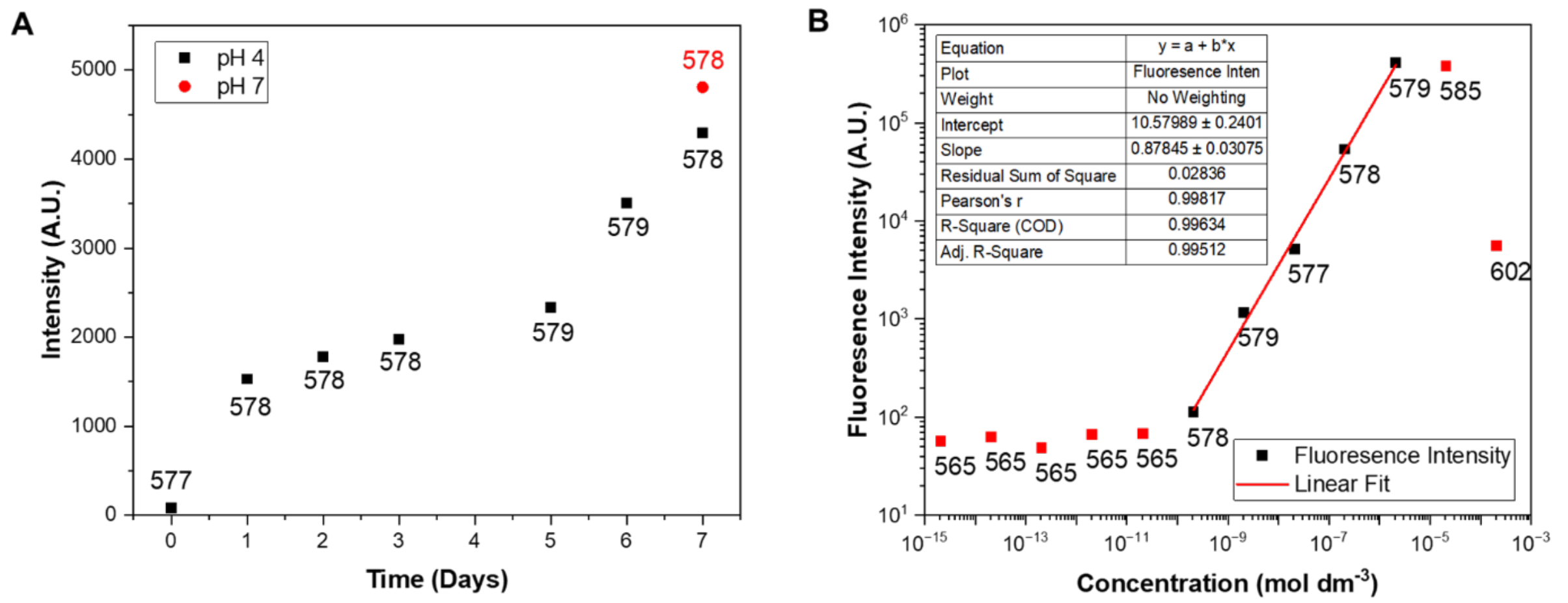

Figure S17 (**A**) Recorded fluorescence intensity of aliquots of dialysis media at pH 4 and7 over time. (**B**) **Rb** calibration curve. Peak maximum wavelengths (cm^{-1}) recorded and displayed adjacent to each datapoint.

5. References

1. T. C. Wong, L. R. Collazo and F. S. Guziec, *Tetrahedron*, 1995, **51**, 649-656.
2. J. A. Jarvis, I. M. Haies, P. T. Williamson and M. Carravetta, *Phys. Chem. Chem. Phys.*, 2013, **15**, 7613-7620.

3. S. Jordans, S. Jenko-Kokalj, N. M. Kühl, S. Tedelind, W. Sendt, D. Brömme, D. Turk and K. Brix, *BMC Biochem*, 2009, **10**, 23.
4. J. Tanaka, A. Evans, P. Gurnani, A. Kerr and P. Wilson, *Polym. Chem.*, 2020, **11**, 2519-2531.
